# Gene expression prediction using low-rank matrix completion

**DOI:** 10.1186/s12859-016-1106-6

**Published:** 2016-06-17

**Authors:** Arnav Kapur, Kshitij Marwah, Gil Alterovitz

**Affiliations:** Biomedical Cybernetics Laboratory, Harvard Medical School, Boston, 02115 MA USA; Department of Health Science and Technology, Electrical Engineering and Computer Science, Massachusetts Institute of Technology, Cambridge, 02139 MA USA

**Keywords:** Prediction, Machine learning, Gene expression

## Abstract

**Background:**

An exponential growth of high-throughput biological information and data has occurred in the past decade, supported by technologies, such as microarrays and RNA-Seq. Most data generated using such methods are used to encode large amounts of rich information, and determine diagnostic and prognostic biomarkers. Although data storage costs have reduced, process of capturing data using aforementioned technologies is still expensive. Moreover, the time required for the assay, from sample preparation to raw value measurement is excessive (in the order of days). There is an opportunity to reduce both the cost and time for generating such expression datasets.

**Results:**

We propose a framework in which complete gene expression values can be reliably predicted in-silico from partial measurements. This is achieved by modelling expression data as a low-rank matrix and then applying recently discovered techniques of matrix completion by using nonlinear convex optimisation. We evaluated prediction of gene expression data based on 133 studies, sourced from a combined total of 10,921 samples. It is shown that such datasets can be constructed with a low relative error even at high missing value rates (>50 %), and that such predicted datasets can be reliably used as surrogates for further analysis.

**Conclusion:**

This method has potentially far-reaching applications including how bio-medical data is sourced and generated, and transcriptomic prediction by optimisation. We show that gene expression data can be computationally constructed, thereby potentially reducing the costs of gene expression profiling. In conclusion, this method shows great promise of opening new avenues in research on low-rank matrix completion in biological sciences.

**Electronic supplementary material:**

The online version of this article (doi:10.1186/s12859-016-1106-6) contains supplementary material, which is available to authorized users.

## Background

A tremendous growth in biomedical information and datasets has been observed in the last two decades [1]. This growth is supported by the development of new technologies that profile gene expressions in an automated manner. Such technologies have significantly evolved in the past 20 years, from initially monitoring less than 50 features per slide [2] to whole genome expression analysis with new generation microarrays having more than 10^6^ features, such as GeneChip oligonucleotide probe based arrays and high density bead arrays [3]. This evolution has persisted in the form of next-generation sequencing (NGS) methods being used to quantify RNA in a sample [4] and have proven to be advantageous in terms of performing discovery-based experiments and having a larger dynamic range.

However, there are fundamental impediments of current profiling technology and gene expression analysis methods. We list a few:

- The cost of commercial RNA-seq and microarray services remain prohibitive and limits their wider adoption in research and clinical applications alike.
- There is a challenge in data storage requirements and high analysis complexity that is associated with datasets sourced from next-generation sequencing (NGS) methods.
- Despite microarray experiments being more economical in terms of cost and data volume, missing data is an inevitable phenomenon in such experiments, and adversely affects downstream analysis. The prevailing missing value imputation algorithms successfully recover expression levels albeit at low missing value rates (only up to 15 % of the expression values).

As of 2015, commercial microarray services cost approximately $450 per sample, and prices vary for different platforms [5–7]. Profiling is generally performed using multiple tests to increase the statistical power of the measurement [8], thus increasing the combined cost of the experiment. The MammaPrint test, a microarray based gene expression test used to predict the risk of recurrence in patients with breast cancer, costs approximately $4,200. Similarly, the Oncotype DX costs more than $3,000 [9]. RNA-Seq is even more expensive than conventional DNA microarray based tests used for gene expression measurements. The cost of RNA sequencing services directly increases with number of reads per sample [10]. There is an upward trend to increase the capacity of such platforms, with manufacturers pushing for higher number of reads and probes per sample, inadvertently increasing the cost per sample. We explore if there is merit to this surge in number of reads and probes to create high dimensional gene expression datasets. For gene expression profiling experiments, it is often the case that a new experiment is designed and performed to capture any novel aspect of interest. We explore a potential possibility of modelling already sourced datasets, and extrapolating these in-silico to discover expression levels of interest.

In this paper, we propose a computational framework to estimate gene expression data using only a selected fraction of gene expression measurements. We demonstrate that the expression levels of certain genes selected from the collection of genes of interest can be used to accurately estimate the remaining expression levels. We show that conclusions regarding expression levels can be derived from partial measurements. We also show that further analysis can be performed using such predicted data, thus enabling the conduction of whole genome expression analysis, using such data. This framework allows for customisation because selected genes can be isolated for analysis. We believe that this method has applications in how biomedical data is sourced and in turn is relevant in the areas of differential gene analysis (class comparison), class prediction, cancer investigation, and non-invasive diagnosis.

## Benefits and contributions

In summary, our key contributions are:

- We demonstrate that gene expression data can be modelled as an approximate low-rank data matrix, in order to computationally predict expression values.
- We show that sparse gene expression measurements (“known” expression levels) could be used to artificially construct the gene expression dataset using non-linear convex optimisation, and report prediction results on diverse expression datasets sourced from multiple experiments. This is in contrast with current biochemical methods which directly measure all expression values.
- We conduct differential gene analysis and Bayesian network analysis on predicted datasets, and compare our results with those obtained using original datasets, to show that the prediction capabilities of the reconstructed and the original datasets are not significantly different.

These technical contributions lead to application areas:

- This can be used to computationally predict behaviour of genes subject to a condition, given a set of measurements. This also has potential applications in consolidating multiple datasets with common phenotypes to infer new transcriptomic behaviour, using low-rank prediction.
- This framework allows for construction of expression datasets using a fraction of known values thereby reducing the number of measurements (in terms of number of probes and reads) required to capture such data.
- We believe that these techniques can potentially reduce the cost of experiments, thus saving millions of dollars, and open a new avenue for research on data completion in other domains, where the observable data is scarce.
- This has applications in high dimensional expression data compression and reconstruction, and can be used to impute missing gene expression data even at high missing value rates.

## Related work

**Biological data and machine learning** Plenty of biological data has generated a need for computational methods to extract useful knowledge from such heterogeneous information. This has led to advancements in machine learning techniques in making predictions particularly applied to data involving proteomics, genomics, and microarrays [11]. Computational models have been successfully used in gene finding [12–14] and prediction of proteins with a secondary structure [15, 16]. More recently, Alipanahi et al. used advancements in deep learning to predict DNA and RNA binding proteins [17]. In the case of expression data, Bayesian networks are effective in modelling relationships between expression profiles for prognosis prediction [18] and inference [19]. Machine learning techniques have been extensively used in expression pattern identification [20, 21] classification [22, 23], and network analysis of expression data [24]. However, the process of measuring expression levels and generating profiles is primarily devoid of any considerable learning or the use of optimisation.

**Low-rank matrix recovery** The objective of recovering a low-rank matrix from a few data samples can be described as an optimisation problem. This is used in various practical scenarios and is a motivation for this study. The Netflix problem is a popular example of how such techniques are applied to recommendation systems [25]. The user–movie data matrix in this case consists of movie ratings (integral values of 1–5) provided by different users for various movies. Because users tend to rate very few movies, the entries in the matrix are sparsely filled. Predicting movie ratings based on such data is used to recommend other movies to the user by posing it as a collaborative filtering problem [26]. The user–movie matrix is assumed to be a low-rank matrix because each movie has a few linearly independent parameters on which the users generally rate the movie. Therefore, only a few samples can be used to predict all the values in the rating matrix.

Low-rank modelling has been applied to computer vision [27] to improve face recognition methods and has been used in novel camera architecture to create high-resolution light fields from a single coded image [28]. In 2003, Basri and Jocobs assumed their high-dimensional image data of convex Lambertian surfaces under different lighting illuminations to exist in a low-dimensional subspace [29]. The concept of low dimensionality has been used to improve background subtraction [30] and motion segmentation [31]. In addition, low-rank matrix recovery is applied for estimating the distance matrix in a triangulation problem when the data available is partial [32, 33].

**Gene expression prediction** In 2004, Nir Friedman proposed a model for predicting gene expression levels by using probabilistic graphical models [34]. Although the method is robust, the performance of accurate prediction is moderate. Approaches involving the information theory [35] have been proposed to identify transcriptional interactions between genes in microarray data, which are computationally inexpensive. However, these approaches do not accurately estimate the expression levels. Methods for estimating missing values in large dimensional expression data are available. For example, the least square imputation method, LL Simpute, involves the combination of similar genes and selects a gene of interest by using k-nearest neighbours [36]. Oba et al. used Bayesian principal component analysis, BPCA, to estimate the missing values in expression profiles [37]. The prevailing methods estimate the gene expression values at very high observabilities of data, that is, unknown values predicted using these methods are extremely few (only up to 10 % of the values). To the best of our knowledge, missing rates of 5 %–10 % are considered moderate and those more than 15 % affect prediction and interpretation [38, 39]. In this study, we attempt to predict high-dimensional expression matrices with only sparse data, with as high as 90 % of the data unknown.

## Methods

In this section, we introduce the principals involved in modeling low-rank matrix completion and artificial construction of the gene expression dataset from known sparse expression levels. We further analyse parameters to improve the prediction performance.

### Model

A gene expression study yields measurements of mRNA levels that represent gene expression values under contrasting experimental conditions, and experiments on multiple samples are consolidated to form a gene expression data matrix. We propose approaching the problem of prediction as recovery from known values as distributed entries in this data matrix. The yet unknown values constitute the complete matrix. The expression data to be predicted can be represented as *M*_*m*×*n*_, where *m* and *n* describe the genes and sample instances respectively. The locations of the known values in the data matrix, also referred to as checkpoint expression values hereafter, are encoded in *Ω*, where (*i,j*)∈*Ω* if expression value is hitherto known.

The proposed framework is an underdetermined system, since the number of measurements is considerably lesser than the number of unknowns. A matrix can be recovered directly by minimising the rank of the data matrix subjected to a certain constraint with the assumption that the data matrix is a low-rank matrix. Ideally, solving the following convex optimisation problem would provide a low-rank matrix that would fit the observed (*i,j*)∈*Ω* entries and recover *M*:

1 $$ \begin{aligned} min &(rank(X)) \\ \text{when } & {X}_{(i,j)} = {M}_{(i,j)} \end{aligned}  $$

Unfortunately, the rank minimisation problem is of NP hard complexity and exact solutions of the problem take doubly exponential computation time, thus rendering the approach impractical for use [40]. It can be shown that the rank minimisation can be remodelled as minimising the sum of the singular values of the data matrix *X*. This is because a matrix with a rank *r* has *r* nonzero singular values, and minimising the rank would essentially be equivalent to minimising the number of nonzero singular values of *X* [32]. This sum is defined as the nuclear norm (Schatten 1-norm or trace norm) of the data matrix:

2 $$  \| X \|* = \sum\limits_{i=1}^{r} \sigma_{i}  $$

where *r* is the rank of *X*, and *σ*_*i*_ is the *i*^*th*^ nonzero singular value of *X*. The nuclear norm is essentially the *l*_1_ norm of the vector of singular values because these values are positive. The decision variable *X* is then heuristically solved as follows:

3 $$  \begin{aligned} min & (\| X \|*) \\ \text{when } & {X}_{(i,j)} = {M}_{(i,j)} \end{aligned}  $$

where (*i,>j*)∈*Ω* the nuclear norm is the tightest convex relaxation of the rank function, and therefore its ideal replacement. The advantage of the nuclear norm is that it is convex, and its global optimum can be efficiently computed. Candès and Recht showed that solution obtained using convex heuristic is the same as that obtained using rank minimisation heuristic, and the replacement holds good under certain conditions [32]. If the predicted gene expression matrix is assumed to be of rank *r*, a lower bound is set on the number of measurements as $|\Omega | \geq Cm^{6/5}r \log m$ for a positive constant *C* and where *m* is the number of distinct genes in the dataset.

### Why low rank?

It is universally known that in any biological process, genes do not act in a solitary manner and rather act in concert [41, 42]. Groups of genes interact in any biological setting, and consequently, the expression levels of genes are interdependent. The association between gene expressions has been studied and analysed in many forms, such as association network structures [24, 43] and pairwise correlations [44]. We believe interdependent factors contribute to the behaviours of transcription factors, thereby influencing the expression of genes and resulting in a highly correlated data matrix. We assume that the gene expression values lie on a low-dimensional linear subspace and the data matrix thus formed may be a low-rank matrix. We later show that this assumption can be considered true to approximately predict these values.

### Expression prediction

The approximate solution to the recovery of the original matrix can be achieved through minimising the nuclear norm. This has gained considerable attention, and various numerical methods are available to solve (3) and obtain the matrix. Biological data is generally characterised by many variables, and high dimensionality of such datasets poses a problem for various numerical methods of recovery. A numerical method used to solve the nuclear norm minimisation problem is to apply a soft-thresholding operation iteratively, which possesses the favourable property of scaling well on large datasets [45]. The following optimisation problem is solved:

4 $$  \begin{aligned} minimise\ \tau \|& X \|* + \frac{1}{2} \| X \|_{F} \\ \text{such that } P_{\Omega} &(X) = P_{\Omega}(M) \end{aligned}  $$

where $ \| A \|_{F} = \sqrt {\sum _{i=1}^{m} \sum _{j=1}^{n} |a_{ij}|^{2}} $ is the Frobenius norm of the matrix, and *P*_*Ω*_ is the orthogonal projection matrix such that:

5 $$  | P_{\Omega} (X)|_{i,j} = \left\{ \begin{array}{ll} X, & \quad (i,j) \in \Omega\\ 0, & \quad (i,j) \notin \Omega\\ \end{array}\right.  $$

Choosing a sufficiently high value of *τ* reduces the influence of the Frobenius norm term in (4), and the optimisation problem described in (4) reduces to the nuclear norm minimisation problem (3), thereby essentially solving for a low-rank matrix. After choosing an appropriate *τ*>0, the expression matrix *X* can be iteratively reconstructed such that the *k*^*th*^ iteration is:

6 $$  \begin{aligned} X^{k} = shrink & \left(Y^{k-1}, \tau\right) \\ Y^{k} = Y^{k-1} + \delta_{k} & {P}_{\Omega} \left(M-X^{k}\right) \end{aligned}  $$

*Y* at *k*=0 is initialised as zero. The shrink is named as the soft thresholding operator [45]. The parameter *τ* determines the amount by which the singular values of the gene data matrix is decreased, thereby determining the rank. The parameter *δ*_*k*_ is the positive step size in the iteration that has been maintained independent of *k*. Therefore, the accuracy of the prediction of expression levels is clearly a strong function of both *τ* and *δ*. The shrink operator can be defined as follows:

7 $$ \begin{aligned} shrink\, (X,\tau) := & \sum\limits_{i=1}^{r}max(\sigma-\tau,0) {u}_{i} {v}_{i}* \\ X= & \sum\limits_{i=1}^{r} \sigma_{i} {u}_{i} {v}_{i}* \end{aligned}  $$

where *u*_*i*_ and *v*_*i*_ are the left singular vectors and right singular vectors of *X*, respectively. The sequence of iterations converges to the desired expression matrix that would minimise (4).

### Parameters

**Fig. 1:**
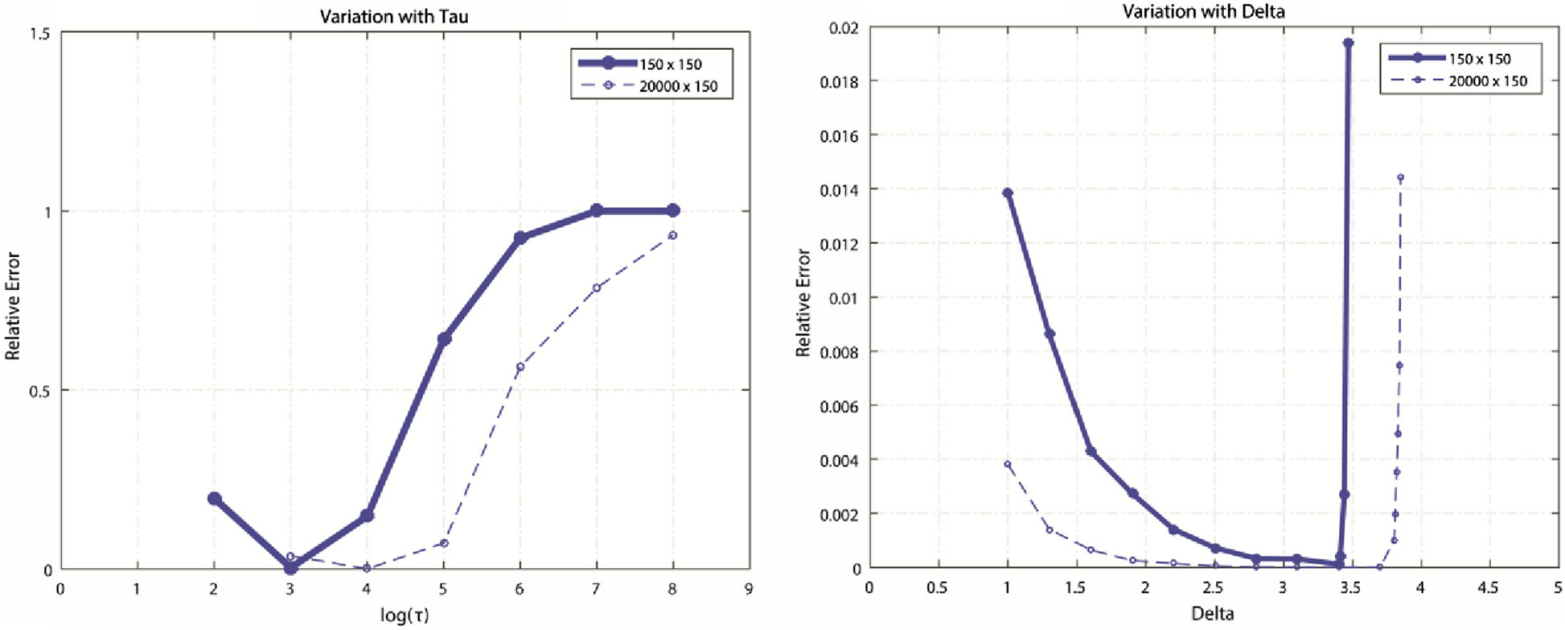
Variation of performance with *τ* and *δ*. This example shows a variation in the relative error in predicting two synthetic datasets of dimensions 150 × 150 and 20000 × 150. The datasets were predicted, and 50 % values were known prior to the prediction at a run of 100 iterations

Notably, the performance of an algorithm depends on the threshold parameter *τ*. High values of *τ* are recommended. However, the question is how high should the parameter be. Selecting an exceedingly high *τ* value may shrink *Y*^*k*^ more than it should, resulting in a low performance. Furthermore, the choice of step size *δ*_*k*_ determines the accuracy of prediction. Incremental changes in the aforementioned parameters can lead to offsets in the performance measures when applied to high-dimensional biomedical datasets. We analysed the variation in the error of prediction on synthetic low-rank matrices of rank 10 (Fig. 1), which were constructed using normally distributed random numbers. In the datasets, 50 % of the values were predicted through low-rank recovery. The values of the parameters responsible for optimal performance depended on the type of data, rank, and size of the datasets. Although optimal parameters can be determined empirically, the following relation could be used [45]:

8 $$  \tau = 5\sqrt{mn}  $$

where dimensions of the final predicted expression matrix are *m* and *n*. The choice of step size for each iteration is a function of known values before prediction [45] and is set as:

9 $$ \delta_{k} = 1.2mn/|\Omega|  $$

The parameters can be further optimised to enhance the prediction performance. To reduce the computation time and the time required for implementations on modest desktop computers, iterations with different values can be performed within a defined range on similar test datasets, pivoted on values determined using (8) and (9). Nevertheless, we demonstrate that the aforementioned relations can be used as is for high accuracy gene expression prediction.

The known checkpoint levels in the gene expression dataset to be predicted are log-transformed before being input for prediction. The expression matrix is then reconstructed iteratively until the error in the convergence of the known expression levels is lower than a threshold tolerance:

10 $$ \frac{||P_{\Omega} \left(X^{k}-M\right) ||_{F}}{||P_{\Omega}(M)||_{F}} \leq \varepsilon  $$

The convergence criterion was empirically set. In our implementation, the tolerance in the error of expression levels was maintained at 10^−8^. An upper limit of the number of iterations was contingent on the available computational power, which was set to 750 iterations.

### Robustness to noise

Gene expression datasets are known to have technical noise in expression level measurements owing to factors such as process errors, lane-to-lane variability in RNA-seq [46, 47] and small sizes of spots, inconsistency in hybridisation, and varying degrees of uniformity of printing pins in microarrays [48–50]. Although, there has been considerable progress in de-noising methods leading to improved expression estimation, and studies show that magnitude of technical noise might be considerably lower than critical levels [51, 52], we evaluated the robustness of the method to noisy datasets. The known checkpoint expression levels can be represented as:

11 $$ x^{'}_{ij} = x_{ij} + \varepsilon_{ij}, (i,j) \in \Omega  $$

where *x*_*ij*_ is the actual value, and *ε*_*ij*_ is the white Gaussian noise term sampled from a distribution with zero mean and standard deviation *σ*_*ε*_. We performed low-rank prediction on synthetic data, which simulated expression data, and varied the standard deviation of the distribution of the additive noise data:

12 $$  {noise\ deviation\ ratio} = \sigma_{\varepsilon}/\sigma_{x}  $$

where *σ*_*x*_ is the standard deviation of the actual set of values. The analysis (Table 1) demonstrated the ability of the method to recover low-rank synthetic noisy data with a low error. We present low-rank prediction results on real gene expression data in the results section.

**Table 1:** Prediction results with additive noise

Ratio	Observability (%)	Relative error
0.003	50	4.22 ×10^−4^
0.03	50	4.21 ×10^−3^
0.3	50	1.78 ×10^−2^
0.003	10	1.21 ×10^−2^
0.03	10	1.57 ×10^−2^
0.3	10	1.91 ×10^−1^

Analysis of the addition of noise to synthetic 2000 × 2000 data matrix of rank 10 in low-rank prediction after 100 iterations

Abbreviations: *Ratio* noise deviation ratio

### Data pre-processing

Data pre-processing can often lead to significant improvement in model performance, and is therefore an imperative step, with normalisation and transformation characteristic to gene expression analysis. The input gene expression data was log-transformed prior to prediction. The distribution of gene expression measurements is heavily skewed, and the values are better correlated after log-transformation, increasing accuracy of low-rank recovery. A variety of normalisation techniques exist for gene expression data analysis, with no clear consensus on a singular strategy. The performance of prediction is enhanced after normalisation; for example, the prediction accuracy with Robust Multi-array Average (RMA) on microarray expression datasets and transforming RNA-seq raw reads into Reads Per Kilobase of transcript per Million mapped reads (RPKM) has a higher prediction accuracy, as compared to prediction performed using raw values. Although, the range of normalisation approaches would be qualified in the case of very low observability of the expression data, data pre-processing with normalisation and transformation is highly recommended for superior results.

## Results and discussion

We present the results of the method in two major parts. First, we evaluated the prediction accuracy on real expression data by using low-rank recovery. Second, we verified whether this predicted dataset can be used as a surrogate of the original dataset for further analysis. We answered this by comparing the results of differential expression analysis obtained using predicted datasets with those obtained using original datasets. Finally, we used Bayesian network modelling for both groups of datasets and compared their results to further address the question.

### Gene expression prediction

The method was evaluated using microarray and RNA-seq based gene expression datasets obtained from the NCBI Gene Expression Omnibus [53] and ArrayExpress [54]. To make the evaluation extensive, we sampled diverse datasets from the repositories. The datasets were diverse in terms of varying number of genes, number of samples, and platforms (see Additional file 1). These datasets were sourced from studies that differed with respect to design of the experiment and measurement approach; examples include disease state using expression arrays, knockdown and knockout experiments using expression arrays, co-expression experiment using RNA-seq of coding RNA, cell type comparison using RNA-seq from single cell amongst others. The observability of an expression dataset quantifies the number of expression values in the data matrix known to the algorithm before prediction as a fraction of the total number of expression values, and thus it can be defined as:

13 $$  O = |\Omega|/mn  $$

**Fig. 2:**
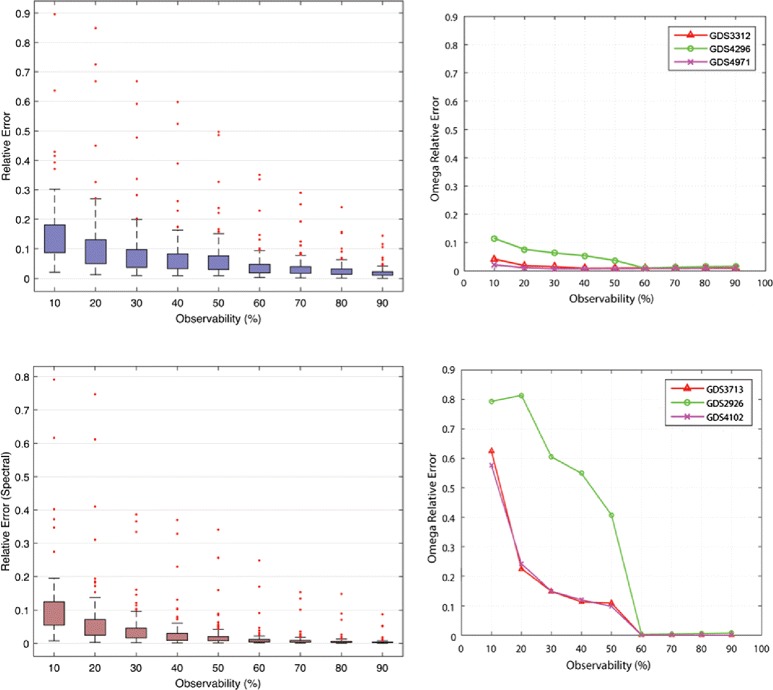
The results of low-rank prediction in 119 datasets containing a combined total of 10,024 microarray slides at 750 iterations. Boxplots representing Frobenius relative error (*top left*) and spectral relative error (*bottom left*) in prediction of converged datasets, and the fraction of values known prior to prediction were varied. Edges of box represent 25 % and 75 % coverage, and the whiskers extend it to 99.73 % coverage, where outliers represent matrices generated using 10 datasets. Variation of omega relative error with the observability of three example datasets with a low Frobenius error (*top right*) and high Frobenius error (*bottom right*). Datasets with a high relative error in prediction (*bottom right*) have a corresponding high omega relative error

For each gene expression dataset, we removed a certain fraction of the total expression values. We created nine incomplete data matrices per dataset with the removal of varied portions of data (10 –90 %) and estimated the expression values at different observabilities. The checkpoint expression levels were selected randomly on the basis of assumptions that the locations were distributed. The error in prediction was averaged over 10 runs of the method for each data matrix, with the locations of checkpoint values in each run being different. We report prediction results on 10,921 samples based on 133 studies (Fig. 2, Additional file 1). The error was assessed by comparing the predicted expression values with the original values by using:

14 $$ \text{Relative Error (Frobenius)} = \|M-X\|_{F}/\|M\|_{F}  $$

15 $$ \text{Relative Error (Spectral)} = \|M-X\|/\|M\|  $$

where *M* and *X* are the original and recovered expression matrices, respectively.

According to the results of the prediction, the expression datasets can be assessed even using reduced measurements (Fig. 2) of the original dataset otherwise generated using high-density commercial array platforms and deep sequencing platforms. Gene expression datasets were predicted using a desktop computer at a standard processing power. The datasets that were predicted can be roughly organised into three major groups, convergence with low error, convergence with high error, and datasets that diverged. The question is how does one separate artificially constructed datasets that converged with a significant low error during the experiment, from the datasets that had a significant error in prediction. The error in convergence of known checkpoint expression values indicated a relative error in predicting the complete dataset (omega error), and this facilitated the determination of the convergence of low-rank completion:

16 $$  \text{Omega Error} = \frac{||P_{\Omega}\left(X^{k}-M\right)||_{F}}{||P_{\Omega}(M)||_{F}}  $$

where *P*_*Ω*_ is the orthogonal projection matrix.

For the same number of iterations in the prediction algorithm, the predicted datasets that converged with a low relative error had a corresponding low omega error, and vice versa for outlier datasets with a high relative error (Fig. 2). Therefore, the error in the convergence of checkpoint expression levels can be used as an indicator of the extent to which predicted expression levels coincide with real values (measured using high-density arrays and RNA-seq). Cross-validation using hold-out rows and columns on a single dataset, and sophisticated methods using weighted Non-negative Matrix Factorisations would give further insight into prediction accuracy [55]. The datasets that did not converge and therefore were not constructed were detected using the omega error.

### Differential expression analysis

We attempt to replicate gene expression profiling experiments using partial measurements, and predicted expression levels basis on these measurements. We identified differentially expressed unique genes by using datasets predicted through low-rank completion and compared the results with those obtained using the original dataset. We also append differential analysis results solely on observed measurements without any prediction or learning to highlight the advantage of such prediction methods.

For instance, we considered a dataset comprising 85 pairs of lesional and non-lesional skin samples with matched biopsies from a patient cohort with moderate to severe psoriasis vulgaris [56] and determined differentially expressed genes (Table 2). On repeating the analysis of artificially constructed datasets by using low-rank completion, the results revealed that the genes differentially expressed in these datasets strikingly overlapped with those identified in the corresponding original datasets. This appears to be true even at low observabilities (see Additional file 1: Tables S1 and S2). The analysis conducted using a predicted psoriasis dataset at 60 % observability (omega relative error = 0.0014, Frobenius relative error = 0.0354, and Spectral relative error = 0.0076) identified S100A12, SERPINB4, SPRR2C, S100A74, KYNU, and TMPRSS11D as the top genes with increased expression in lesional skin compared with corresponding non-lesional samples (*P*-values ranging from 10^−58^ to 10^−46^), and this result is consistent with those reported previously [56].

**Table 2:** Differential analysis on predicted expression datasets. Top unique differentially expressed genes upregulated in lesional skin compared with those in non-lesional skin when ranked according to log2-fold-change in (a) original dataset, (b) predicted dataset with 60 % observability and (c) sparse known-value (checkpoint) dataset without prediction at 60 % observability

		Original dataset				Recovered dataset (60 %)				Checkpoint dataset (60 %)		
Gene	Probe ID	Symbol FC	log	Adj.	Probe ID	Symbol	log FC	Adj.	Probe ID	Symbol	log FC	Adj.
ranking				*P*-Value ×10^−10^				*P*-Value ×10^−10^				*P*-Value
1	205863_at	S100A12	9.79929	< 1	205863_at	S100A12	8.99648	< 1	211906_s_at	SERPINB4	6.21118	3.3×10^−10^
2	211906_s_at	SERPINB4	9.60376	< 1	211906_s_at	SERPINB4	8.67119	< 1	205863_at	S100A12	5.48282	3.3×10^−9^
3	205513_at	TCN1	8.65788	< 1	205513_at	TCN1	8.12271	< 1	205513_at	TCN1	5.07988	4.8×10^−9^
4	232220_at	S100A7A	8.21988	< 1	232220_at	S100A7A	7.92112	< 1	204385_at	KYNU	5.06729	3.3×10^−10^
5	205660_at	OASL	7.94647	< 1	205660_at	OASL	7.4045	< 1	1569555_at	GDA	4.75835	4.8×10^−9^
6	220664_at	SPRR2C	7.87929	< 1	220664_at	SPRR2C	7.3366	< 1	205844_at	VNN1	4.70129	3.3×10^−10^
7	207602_at	TMPRSS11D	7.64471	< 1	1569555_at	GDA	7.11896	< 1	209719_at	SERPINB3	4.67529	1.6×10^−4^
8	1569555_at	GDA	7.39506	< 1	207602_at	TMPRSS11D	7.10503	< 1	234699_at	RNASE7	4.57012	2.9×10^−7^

Significance is demonstrated by adjusted *P*-values for fold change in every gene by using eBayes with Benjamini–Hochberg correction

Abbreviations: *logFC* log2-fold-change, *Ave Expr* average log2-expression of the probe over all arrays, *Adj. P-Value* *P*-value adjusted from the raw *P*-value

**Fig. 3:**
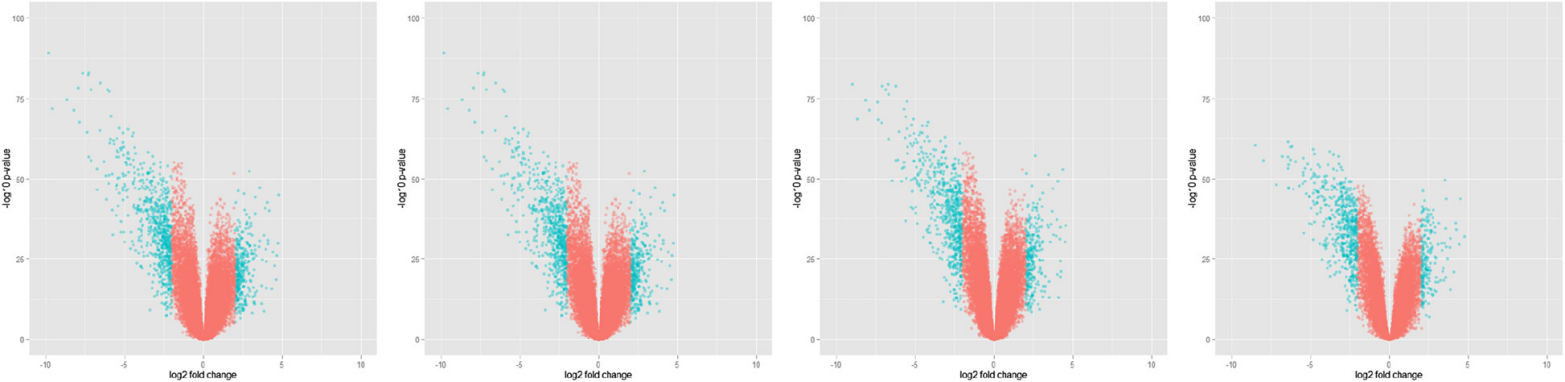
Comparison of differential analysis on original and predicted datasets. Volcano plots represent differentially expressed genes at logFC > 2 and FDR P < 0.05 in original psoriasis vulgaris dataset (*leftmost*), predicted dataset with 10 % values unknown, with 40 % values unknown and with 70 % values unknown (*rightmost*)

Similarly, in another expression profiling experiment, the oral mucosa of smokers was compared with that of nonsmokers [57], and the top genes were identified using the predicted dataset at 50 % observability (omega relative error = 0.0412, Frobenius relative error = 0.0404, and spectral relative error = 0.0073). The genes ranked according to the fold change were CYP1B1, S100A7, KRT76, RPTN, and PNLIPRP3 (false discovery rate, FDR = 0.05; *P*-value =10^−5^ to 10^−2^). The results were consistent with those reported previously [57]. The entire list and comparison is described in Additional file 1: Table S2. We obtained similar results for the differential analysis conducted in other studies (Additional file 1). The results indicated that differentially expressed genes with sparse gene expression measurements and incomplete expression data can be identified. The degree to which the predicted datasets emulate the behaviour of the original dataset increases with an increase in the observability at the time of prediction (Fig. 3).

### Probabilistic modelling and classification

The problem of the classification of samples into biological classes of tissues and diseases has been a crucial topic of research. We explored the possibility of using data that is artificially constructed to train the classifier instead of the original gene expression data. We used Bayesian networks for modelling the expression levels of genes and class prediction. Bayesian networks provide a means to model the stochastic nature of biological data and capture causal relationships between expression levels of genes for inference on new unseen data and for classifying owing to high prediction accuracies [58, 59].

The analysis comprised many steps. We first preprocessed both groups of datasets by discretising the gene expression levels into three states, underexpressed, baseline, and overexpressed [58]. We trained Bayesian network classifiers on reduced datasets of 100 gene variables from the predicted datasets and the corresponding original datasets to shrink the search space of dependent networks. Classification accuracy was determined in a multiple run 10 fold cross validation analysis. We include comparison of Bayesian network classification trained on microarray datasets of lung adenocarcinoma [60], myelodysplastic syndrome [61], pancreatic ductal adenocarcinoma [62], psoriasis [56], pulmonary fibrosis [63] with corresponding low-rank predicted datasets and datasets sampled from a uniform distribution. The performances of the classifiers obtained using low-rank recovered datasets matched with those of classifiers obtained using corresponding original datasets (Table 4). Furthermore, we compared the class predictions and probability distributions of individual test instances (see Additional file 1: Table S4). In this section, we presented the results at low observabilities to demonstrate lower bound cases.

We also verify whether data predicted using low-rank matrix completion performed superior to data built using sampled values from a known distribution, and whether high performance of low-rank predicted datasets is just due to the known checkpoint expression levels. In this third group of datasets, the expression levels were populated by sampling values from a uniform distribution between the maximum and minimum expression levels of the data known before prediction instead of low-rank recovery. The analysis was repeated for these datasets, and the classifier prediction results were compared with the classifier learned on original datasets (Table 3 and see Additional file 1: Tables S4). The classifiers trained using these datasets had a distinctly lower performance than classifiers trained using low-rank predicted and original datasets.

**Table 3:** Top unique differentially expressed genes upregulated in lesional skin compared with those in non-lesional skin when ranked according to log2-fold-change in (a) original dataset, (b) predicted dataset with 30 % observability, and (c) sparse known-value (checkpoint) dataset without prediction at 30 % observability

		Original dataset				Recovered dataset (30 %)				Checkpoint dataset (30 %)		
Gene	Probe ID	Symbol	log FC	Adj.	Probe ID	Symbol	log FC	Adj.	Probe ID	Symbol	log FC	Adj.
ranking				*P*-Value ×10^−10^				*P*-Value ×10^−10^				*P*-Value
1	205863_at	S100A12	9.79929	< 1	205863_at	S100A12	8.48947	< 1	207367_at	ATP12A	3.17871	0.02
2	211906_s_at	SERPINB4	9.60376	< 1	211906_s_at	SERPINB4	7.98211	< 1	201086_x_at	SON	3.12259	0.17
3	205513_at	TCN1	8.65788	< 1	220664_at	SPRR2C	7.17109	< 1	213356_x_at	NA	3.06212	0.29
4	232220_at	S100A7A	8.21988	< 1	232220_at	S100A7A	6.77508	< 1	209719_x_at	SERPINB3	2.98365	0.15
5	205660_at	OASL	7.94647	< 1	204385_at	KYNU	6.4279	< 1	33322_i_at	SFN	2.89353	0.36
6	220664_at	SPRR2C	7.87929	< 1	207602_at	TMPRSS11D	6.41765	< 1	213523_at	KIAA0368	2.88306	0.29
7	207602_at	TMPRSS11D	7.64471	< 1	207367_at	ATP12A	6.40415	< 1	210413_x_at	CCNE1	2.83059	0.06
8	1569555_at	GDA	7.39506	< 1	210413_x_at	NA	6.39934	< 1	217388_s_at	NA	2.82118	0.19

It is to be noted that the analysis performed solely on known expression values (c) gives incorrect conclusions. However, the results of analysis after low-rank prediction matched with those obtained using original dataset

Abbreviations: *logFC* log2-fold-change, *Ave Expr* average log2-expression of the probe over all arrays, *Adj. P-Value* *P*-value adjusted from the raw *P*-value

**Table 4:** Comparison of the results of classification obtained using Bayesian networks learnt on low observability predicted datasets with those in which networks were learnt on original datasets

Study	Dataset	True positive rate	False positive rate	Precision	Recall	F-measure	AUROC
Lung adenocarcinoma	Original	0.944	0.057	0.944	0.944	0.944	0.988
	Low-rank prediction	0.944	0.057	0.944	0.944	0.944	0.996
	(*O* = 60 %)						
	Sampled Uniform distribution	0.757	0.256	0.758	0.757	0.755	0.777
	(*O* = 60 %)						
Myelodysplastic syndrome	Original	0.865	0.866	0.844	0.865	0.854	0.673
	Low-rank prediction	0.865	0.92	0.833	0.865	0.849	0.675
	(*O* = 40 %)						
	Sampled Uniform distribution	0.85	0.868	0.842	0.85	0.846	0.425
	(*O* = 40 %)						
Pulmonary hypertension	Original	0.638	0.121	0.633	0.638	0.635	0.854
	Low-rank prediction	0.681	0.118	0.645	0.681	0.659	0.897
	(*O* = 60 %)						
	Sampled Uniform distribution	0.267	0.372	0.213	0.267	0.218	0.424
	(*O* = 60 %)						
Pancreatic ductal	Original	0.782	0.218	0.784	0.782	0.782	0.886
adenocarcinoma	Low-rank prediction	0.821	0.179	0.821	0.821	0.82	0.905
	(*O* = 50 %)						
	Sampled Uniform distribution	0.397	0.603	0.389	0.397	0.385	0.417
	(*O* = 50 %)						
Psoriasis	Original	0.912	0.088	0.913	0.912	0.912	0.96
	Low-rank prediction	0.912	0.088	0.912	0.912	0.912	0.956
	(*O* = 40 %)						
	Sampled Uniform distribution	0.641	0.359	0.641	0.641	0.641	0.648
	(*O* = 40 %)						

Datasets were condensed and constituted of randomly selected 100 gene attributes. Bayesian networks were learned using a bottom-up search method known as K2 algorithm and evaluated in a 10-fold cross validation analysis. The predicted datasets were evaluated by comparing the classification results with those obtained using datasets constructed employing values sampled from a set uniform distribution instead of low-rank recovery, and the fraction of known values were the same in both cases. Notably, the performance of low-rank recovered datasets closely matched with that of the original datasets

Abbreviations: *O* observability, *AUROC* Area Under the Receiver Operating Characteristic curve deviation ratio

The results indicated that Bayesian networks constructed using low-rank recovered datasets closely resemble those constructed using original datasets, irrespective of classifier accuracy. For instance, the area under the receiver operating characteristic curve (AUROC) of the network constructed using the original and predicted Myelodysplastic syndrome datasets were 0.673 and 0.675 (Table 3, *P*-value < 0.01), respectively, whereas the AUROC of the original and predicted pulmonary hypertension dataset were 0.854 and 0.897 (Table 3, *P*-value < 0.001), respectively.

## Conclusions

In this article, we described the modelling of biological datasets as low-rank matrices subject to their inherent dependencies. These datasets can be recovered using the mathematics of low-rank matrix completion. We used random samples as checkpoints. However, quantitatively derived checkpoints can function more satisfactorily than random samples. This provides a foundation for future work in which prediction accuracy, particularly at low observabilities, could be further improved.

Moreover, we see a clear scenario in which such techniques can be applied to other datasets in biomedicine. This framework allows for prediction of biomedical quantities, in likeness to recommender systems, given a set of observable values. Such a framework also has applications in fields in which data collection is precious and prediction could be made using partial measurements. The method can be further developed to manage data volumes sourced from high-throughput sequencing methods. The method can be used as an imputation method, when there is partial data loss as is prevalent in using microarrays today. A major concern in current convex algorithms is the computational requirement. However, datasets with hundreds of millions of points can be accurately predicted using highly parallel processing using GPUs and the cloud.

We believe that this study will open new avenues in research on low-rank matrix completion in biological sciences. We show how much information is inherently present in the actual matrix for gene expression thereby telling us how many measurements we really need to make. We believe biomedical researchers will design actual experiments based on this information opening up new avenues in research on such techniques.

## Abbreviations

AUROC, area under receiver operating curve; BPCA, Bayesian principal component analysis; LLSimpute, local least square imputation; NCBI, National Center for Biotechnology Information; NGS, next generation sequencing; NP, nondeterministic polynomial time; RKPM, reads per kilobase of transcript per million mapped reads; RMA, robust multi-array average

## Additional files

Additional file 1 In this supplement, we provide additional discussion and further analysis on additional studies. (PDF 851 kb)

Additional file 2 In this file, we provide description and sources of studies used in this study. (PDF 516 kb)
