# Supplementary material for: Gene expression prediction using low-rank matrix completion
Source: BMC Bioinformatics. 2016 Jun 17;17:243. doi: 10.1186/s12859-016-1106-6 (PMC4912738; doi:10.1186/s12859-016-1106-6)
Supplement: Additional file 1 — In this supplement, we provide additional discussion and further analysis on additional studies. (PDF 851 kb) [file 12859_2016_1106_MOESM1_ESM.pdf]

Supplementary Material:
Gene Expression Prediction using Low-Rank Matrix Completion

Arnav Kapur[1,*] Kshitij Marwah[1] Gil Alterovitz[1,2]

[1]Biomedical Cybernetics Laboratory, Harvard Medical School, Boston, MA 02115 and [2]Department of Health Science and Technology/Electrical Engineering and Computer Science, Massachusetts Institute of Technology, Cambridge, MA 02139.

Abstract

In this supplement, we describe the source of gene expression data used to evaluate low-rank prediction, provide additional discussion and results on additional studies.

S.1 Gene Expression Data

Our evaluation set consisted of gene expression datasets sourced from the curated expression data (GDS) repository of the Gene Expression Omnibus (NCBI GEO), with samples within a dataset referring to a common Platform, and curated high-throughput sequencing data from ArrayExpress. The expression values of each Sample within a curated dataset were transformed on a log2 scale. Failure of prediction for select datasets was indicated when successive iterates had an increasing error, implying divergence. This was indicated when the Omege error increased beyond a set threshold. The predictions were performed on a Windows ® 8.0 desktop CPU with an Intel ® Core ™ i5-3470 processor (3.40 GHz), and 16 GB RAM. The evaluation set included studies performed on the following microarray platforms:

- Affymetrix Human Genome U95A Array
- Affymetrix Human Genome U95 Version 2 Array
- Affymetrix Human Genome U95B Array
- Affymetrix GeneChip Human Genome HG-U133 Plus 2 Array
- Affymetrix Human Genome U133 Plus 2.0 Array
- Affymetrix Human Genome U133A Array
- Affymetrix Human Genome U133A 2.0 Array
- Affymetrix Human Genome U133B Array
- Affymetrix HT HG-U133+ PM Array Plate
- Affymetrix Human Gene 1.0 ST Array
- Affymetrix Human Gene 1.1 ST Array
- ABI Human Genome Survey Microarray Version 2
- Affymetrix GeneChip Human Genome U133A 2.0 Array
- Agilent-012097 Human 1A Microarray (V2) G4110B
- Agilent-012391 Whole Human Genome Oligo Microarray G4112A
- Illumina HumanHT-12 V3.0 expression beadchip
- Illumina HumanRef-8 v2.0 expression beadchip
- Illumina HumanRef-8 v3.0 expression beadchip
- NCI_UniGEM2_HCC
- NHICU Human 19K v1.0
- NuGO array (human) NuGO_Hs1a520180
- Research Genetics (Invitrogen) - GF211 Microarray Filter
- Rosetta/Merck Human RSTA Custom Affymetrix 2.0 microarray

The protocol hardware for RNA-seq based studies were variants of the Illumina sequencing kits:

- Illumina HiSeq 1500
- Illumina HiSeq 2000
- Illumina HiSeq 2500
- Illumina Genome Analyzer II
- Illumina Genome Analyzer IIx
- Illumina HiScanSQ

S.2 Differential Expression and Bayesian Network Analysis

We present results of identifying differentially expressed genes in three additional studies, and the comparison of the analysis with low-rank predicted and original datasets. Differentially expressed genes were identified in both original and low-rank predicted profiles of acute myeloid leukemia (Stirewalt *et al.*, 2008), non-small cell lung carcinoma (Lu *et al.*, 2010) and the oral mucosa of smokers (Boyle *et al.*, 2010).

For each dataset, we performed the predictions at different observabilities, by randomly selecting the checkpoint expression values at each observability. The genes were then ranked according to log2 fold change in both the original and constructed datasets, using the Bayes moderated t-test.

This supplement includes disease classification results on individual test examples trained with the original Pancreatic Ductal Adenocarcinoma [4] and low-rank predicted dataset with 50% missing values, demonstrating the ability to derive prognostic and diagnostic signatures from low-rank predicted gene expression data.

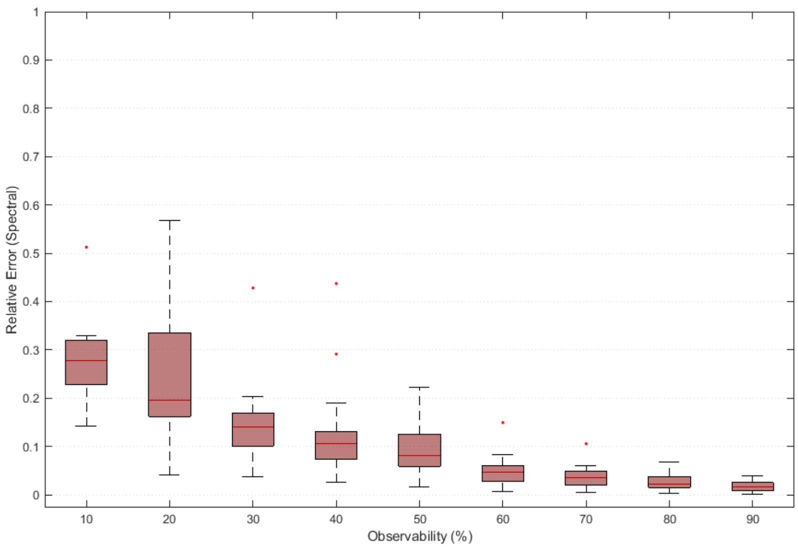

Figure S.1: Boxplot represents spectral relative error in prediction of converged RNA-seq datasets evaluated on a combined total of 897 samples, sourced from 14 gene expression studies. The observability quantifies fraction of measurements known prior to prediction. Each dataset was replicated into 9 equivalents with varying observability (10%-90%). Edges of box represent 25% and 75% coverage and the whiskers extend to 99.3% coverage.

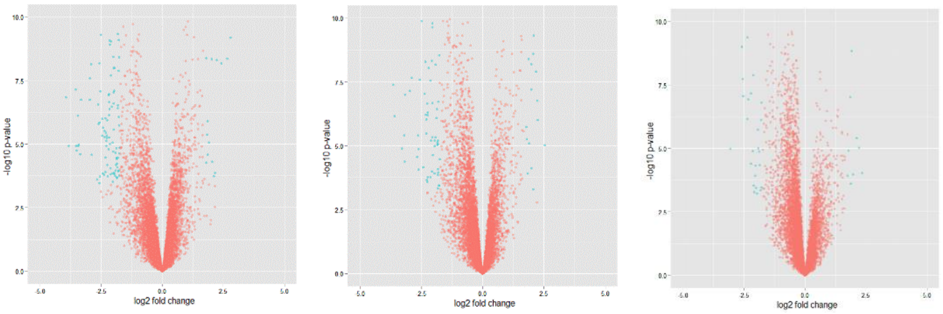

Figure S.2: Comparison of differential analysis on original and predicted acute myeloid leukemia (AML) datasets. Volcano plots represent differentially expressed in original AML dataset (leftmost), low-rank predicted dataset with 40% values unknown, and with 50% values unknown (rightmost).

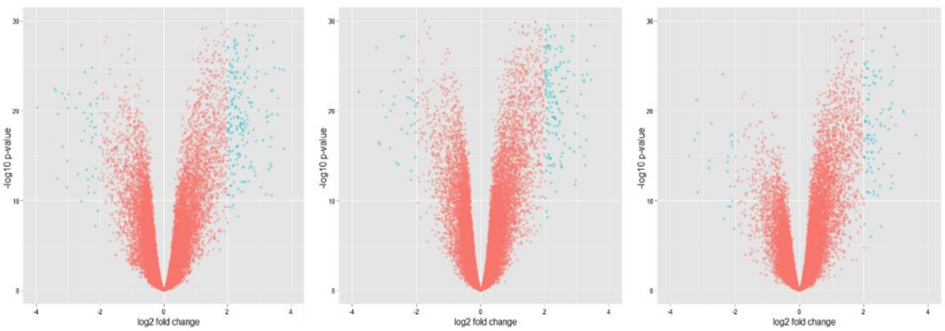

Figure S.3: Comparison of differential analysis on original and predicted non-small cell lung carcinoma (NSCLC) datasets. Volcano plots represent differentially expressed in original NSCLC dataset (leftmost), low-rank predicted dataset with 40% values unknown, and with 70% values unknown (rightmost).

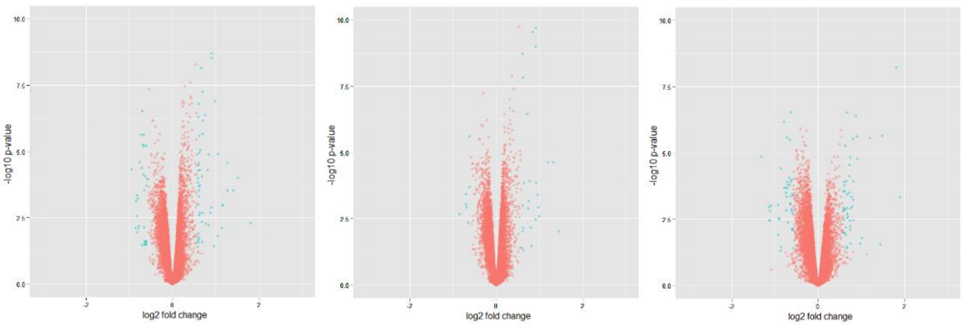

Figure S.4: Comparison of differential analysis on original and predicted oral mucosa datasets. Volcano plots represent differentially expressed in the original oral mucosa of smokers when compared with that of non-smokers (leftmost), low-rank predicted dataset with 40% values unknown, and with 70% values unknown (rightmost).

Table S.1.A: Top unique differentially expressed genes in non-small cell lung carcinoma when ranked according to log2-fold-change in **(a)** original dataset, **(b)** predicted dataset on known checkpoint values with 60% observability, and **(c)** data with known checkpoint values without prediction at 60% observability. Significance is demonstrated by adjusted P-values for fold change in every gene by using eBayes with Benjamini–Hochberg correction.

	Original Dataset				Predicted Dataset (60%)				Checkpoint Dataset (60%)			
Gene Ranking	Probe ID	Symbol	log FC	Adj. P-Value	Probe ID	Symbol	log FC	Adj. P-Value	Probe ID	Symbol	log FC	Adj. P-Value
1.	210081_at	*AGER*	4.397615	$< 1 \times 10^{-10}$	210081_at	*AGER*	4.281678	$< 1 \times 10^{-10}$	228698_at	*SOX7*	3.653878	0.0577
2.	209470_s_at	*GPM6A*	4.198425	$< 1 \times 10^{-10}$	232578_at	*CLDN18*	4.097738	$< 1 \times 10^{-10}$	217414_x_at	*NA*	3.554961	0.3019
3.	232578_at	*CLDN18*	4.160882	$< 1 \times 10^{-10}$	209470_s_at	*GPM6A*	3.789067	$< 1 \times 10^{-10}$	219866_at	*CLIC5*	3.511534	0.0577
4.	206209_s_at	*CA4*	3.869421	$< 1 \times 10^{-10}$	203980_at	*FABP4*	3.541133	$< 1 \times 10^{-10}$	225274_at	*PCYOX1*	3.452415	0.2424
5.	204712_at	*WIF1*	3.773603	$< 1 \times 10^{-10}$	206209_s_at	*CA4*	3.513062	$< 1 \times 10^{-10}$	228766_at	*CD36*	3.418187	0.0891
6.	203980_at	*FABP4*	3.747368	$< 1 \times 10^{-10}$	209469_at	*GPM6A*	3.435685	$< 1 \times 10^{-10}$	230469_at	*RTKN2*	3.375043	0.0003
7.	230560_at	*STXBP6*	3.627475	$< 1 \times 10^{-10}$	230560_at	*STXBP6*	3.424782	$< 1 \times 10^{-10}$	205200_at	*NA*	3.359106	0.2563
8.	209469_at	*GPM6A*	3.615812	$< 1 \times 10^{-10}$	219230_at	*TMEM100*	3.419413	$< 1 \times 10^{-10}$	200902_at	*SEP15*	3.320577	0.2563

Table S.1.B Top unique differentially expressed genes in non-small cell lung carcinoma when ranked according to log2-fold-change in **(a)** original dataset, **(b)** predicted dataset with 30% observability, and **(c)** data with known checkpoint values without prediction at 30% observability.

	Original Dataset				Predicted Dataset (30%)				Checkpoint Dataset (30%)			
Gene Ranking	Probe ID	Symbol	log FC	Adj. P-Value	Probe ID	Symbol	log FC	Adj. P-Value	Probe ID	Symbol	log FC	Adj. P-Value
1.	210081_at	*AGER*	4.397615	$< 1 \times 10^{-10}$	214387_x_at	*SFTPC*	3.615717	$< 1 \times 10^{-10}$	211813_x_at	*DCN*	3.528874	0.000616
2.	209470_s_at	*GPM6A*	4.198425	$< 1 \times 10^{-10}$	230560_at	*STXBP6*	3.239639	$< 1 \times 10^{-10}$	200716_x_at	*NA*	3.259112	0.003868
3.	232578_at	*CLDN18*	4.160882	$< 1 \times 10^{-10}$	210081_at	*AGER*	3.213539	$< 1 \times 10^{-10}$	213048_s_at	*SET*	3.014932	0.002372
4.	206209_s_at	*CA4*	3.869421	$< 1 \times 10^{-10}$	209613_s_at	*ADH1B*	3.086986	$< 1 \times 10^{-10}$	201716_at	*SNX1*	2.922509	0.000319
5.	204712_at	*WIF1*	3.773603	$< 1 \times 10^{-10}$	209074_s_at	*FAM107A*	3.020343	$< 1 \times 10^{-10}$	212537_x_at	*NA*	2.890575	0.008841
6.	203980_at	*FABP4*	3.747368	$< 1 \times 10^{-10}$	217546_at	*MT1M*	2.985399	$< 1 \times 10^{-10}$	202029_x_at	*RPL38*	2.874477	0.008984
7.	230560_at	*STXBP6*	3.627475	$< 1 \times 10^{-10}$	209470_s_at	*GPM6A*	2.943318	$< 1 \times 10^{-10}$	206621_s_at	*EIF4H*	2.853773	0.003253
8.	209469_at	*GPM6A*	3.615812	$< 1 \times 10^{-10}$	242009_at	*SLC6A4*	2.93128	$< 1 \times 10^{-10}$	212294_at	*GNG12*	2.837949	0.000915

Table S.2.A Top unique differentially expressed genes in oral mucosa of smokers when ranked according to log2-fold-change in **(a)** original dataset, **(b)** predicted dataset with 60% observability, and **(c)** data with known checkpoint values without prediction at 60% observability.

	Original Dataset				Predicted Dataset (60%)				Checkpoint Dataset (60%)			
Gene Ranking	Probe ID	Symbol	log FC	Adj. P-Value	Probe ID	Symbol	log FC	Adj. P-Value	Probe ID	Symbol	log FC	Adj. P-Value
1.	202437_s_at	*CYP1B1*	2.18598	$< 1 \times 10^{-10}$	202437_s_at	*CYP1B1*	1.753346	2.06×10^{-10}	226188_at	*LGALSL*	3.595662	0.9215
2.	231628_s_at	*SERPINB6*	1.801147	9.96×10^{-2}	231628_s_at	*SERPINB6*	1.447066	1.26×10^{-1}	219554_at	*RHCG*	3.582851	0.9428
3.	202436_s_at	*CYP1B1*	1.786575	$< 1 \times 10^{-10}$	202436_s_at	*CYP1B1*	1.380508	5.81×10^{-7}	203303_at	*DYNLT3*	3.533785	0.9215
4.	1553454_at	*RPTN*	1.501974	1.44×10^{-2}	1553454_at	*RPTN*	1.329623	8.73×10^{-3}	203534_at	*LSM1*	3.528252	0.6176
5.	205916_at	*S100A7*	1.409739	2.65×10^{-2}	206198_s_at	*CEACAM7*	1.188474	8.73×10^{-3}	212284_x_at	*TPT1*	3.450075	0.9428
6.	202435_s_at	*CYP1B1*	1.333991	5.57×10^{-9}	203290_at	*HLA-DQA1*	0.999711	7.10×10^{-2}	200741_s_at	*RPS27*	3.387568	0.9428
7.	206198_s_at	*CEACAM7*	1.27266	2.68×10^{-2}	207878_at	*KRT76*	0.978685	5.15×10^{-2}	222466_s_at	*MRPL42*	3.383394	0.5249
8.	207720_at	*LOR*	1.269648	6.74×10^{-3}	205916_at	*S100A7*	0.959343	3.08×10^{-2}	209351_at	*KRT14*	3.38324	0.9428

Table S.2.B: Top unique differentially expressed genes in oral mucosa of smokers when ranked according to log2-fold-change in **(a)** original dataset, **(b)** predicted dataset with 40% observability, and **(c)** data with known checkpoint values without prediction at 40% observability.

	Original Dataset				Predicted Dataset (40%)				Checkpoint Dataset (40%)			
Gene Ranking	Probe ID	Symbol	log FC	Adj. P-Value	Probe ID	Symbol	log FC	Adj. P-Value	Probe ID	Symbol	log FC	Adj. P-Value
1.	202437_s_at	*CYP1B1*	2.18598	$< 1 \times 10^{-10}$	1553454_at	*RPTN*	1.887365	4.46×10^{-2}	1553538_s_at	*COX1*	4.100917	0.923525
2.	231628_s_at	*SERPINB6*	1.801147	9.96×10^{-2}	202437_s_at	*CYP1B1*	1.807883	1.61×10^{-4}	221700_s_at	*UBA52*	3.968607	0.70759
3.	202436_s_at	*CYP1B1*	1.786575	$< 1 \times 10^{-10}$	202436_s_at	*CYP1B1*	1.484648	5.17×10^{-3}	210927_x_at	*JTB*	3.898795	0.644659
4.	1553454_at	*RPTN*	1.501974	1.44×10^{-2}	231628_s_at	*SERPINB6*	1.439264	2.30×10^{-1}	1553588_at	*NA*	3.764332	0.978179
5.	205916_at	*S100A7*	1.409739	2.65×10^{-2}	202435_s_at	*CYP1B1*	1.191601	5.17×10^{-3}	218990_s_at	*SPRR3*	3.547704	0.981083
6.	202435_s_at	*CYP1B1*	1.333991	5.57×10^{-9}	204409_s_at	*EIF1AY*	1.12443	3.28×10^{-1}	200024_at	*RPS5*	3.529034	0.973445
7.	206198_s_at	*CEACAM7*	1.27266	2.68×10^{-2}	206198_s_at	*CEACAM7*	1.008266	1.87×10^{-1}	217732_s_at	*ITM2B*	3.484147	0.978179
8.	207720_at	*LOR*	1.269648	6.74×10^{-3}	207356_at	*NA*	0.972234	2.23×10^{-1}	202403_s_at	*COL1A2*	3.42695	0.923525

Table S.3.A: Top unique differentially expressed genes in acute myeloid leukemia when ranked according to log2-fold-change in **(a)** original dataset, **(b)** predicted dataset with 60% observability, and **(c)** data with known checkpoint values without prediction at 60% observability.

	Original Dataset				Predicted Dataset (60%)				Checkpoint Dataset (60%)			
Gene Ranking	Probe ID	Symbol	log FC	Adj. P-Value	Probe ID	Symbol	log FC	Adj. P-Value	Probe ID	Symbol	log FC	Adj. P-Value
1.	215489_x_at	*HOMER3*	2.806378	2.57×10^{-8}	214575_s_at	*AZU1*	2.528969	0.000242	200674_s_at	*RPL32*	4.830232	0.655973
2.	206674_at	*FLT3*	2.79343	2.44×10^{-7}	205131_x_at	*CLEC11A*	2.246066	4.36×10^{-5}	208313_s_at	*SF1*	4.328534	0.603705
3.	210365_at	*NA*	2.704193	4.40×10^{-8}	206674_at	*FLT3*	2.235996	1.19×10^{-5}	203633_at	*CPT1A*	4.317194	0.603705
4.	204647_at	*HOMER3*	2.667401	9.41×10^{-7}	214651_s_at	*NA*	2.208055	0.011649	207783_x_at	*HUWE1*	4.244694	0.769227
5.	205899_at	*CCNA1*	2.415505	1.25×10^{-6}	215489_x_at	*HOMER3*	2.173949	1.60×10^{-6}	208645_s_at	*RPS14*	3.845344	0.783248
6.	206067_s_at	*WT1*	2.337608	6.39×10^{-8}	210365_at	*NA*	2.078888	1.66×10^{-7}	200926_at	*RPS23*	3.731364	0.835709
7.	201105_at	*LGALS1*	2.236192	1.01×10^{-6}	206067_s_at	*WT1*	2.056385	4.78×10^{-7}	211530_x_at	*HLA-G*	3.724911	0.655973
8.	205131_x_at	*CLEC11A*	2.150491	2.11×10^{-3}	205382_s_at	*CFD*	2.053737	0.004836	200775_s_at	*NA*	3.562129	0.769227

Table S.3.B: Top unique differentially expressed genes in acute myeloid leukemia when ranked according to log2-fold-change in **(a)** original dataset, **(b)** predicted dataset with 50% observability, and **(c)** data with known checkpoint values without prediction at 50% observability.

	Original Dataset				Predicted Dataset (50%)				Checkpoint Dataset (50%)			
Gene Ranking	Probe ID	Symbol	log FC	Adj. P-Value	Probe ID	Symbol	log FC	Adj. P-Value	Probe ID	Symbol	log FC	Adj. P-Value
1.	215489_x_at	*HOMER3*	2.806378	2.57×10^{-8}	214651_s_at	*NA*	2.350099	9.25×10^{-5}	200610_s_at	*NCL*	5.909409	0.110193
2.	206674_at	*FLT3*	2.79343	2.44×10^{-7}	209905_at	*NA*	2.203104	9.67×10^{-6}	209191_at	*TUBB6*	4.111017	0.24289
3.	210365_at	*NA*	2.704193	4.40×10^{-8}	210549_s_at	*CCL23*	2.115887	3.93×10^{-6}	200088_x_at	*NA*	3.990415	0.768825
4.	204647_at	*HOMER3*	2.667401	9.41×10^{-7}	209560_s_at	*DLK1*	1.957567	1.05×10^{-4}	201144_s_at	*EIF2S1*	3.921335	0.315863
5.	205899_at	*CCNA1*	2.415505	1.25×10^{-6}	215489_x_at	*HOMER3*	1.901405	1.41×10^{-9}	201406_at	*NA*	3.738085	0.797762
6.	206067_s_at	*WT1*	2.337608	6.39×10^{-8}	217996_at	*PHLDA1*	1.86339	2.5×10^{-4}	217907_at	*MRPL18*	3.687096	0.315863
7.	201105_at	*LGALS1*	2.236192	1.01×10^{-6}	204647_at	*HOMER3*	1.84943	9.62×10^{-8}	221766_s_at	*FAM46A*	3.659216	0.713662
8.	205131_x_at	*CLEC11A*	2.150491	2.11×10^{-3}	210365_at	*NA*	1.784583	1.23×10^{-5}	200961_at	*SEPHS2*	3.580836	0.315863

Table S.4: Comparison of class predictions and probability distributions of Bayesian networks built by employing an original pancreatic ductal adenocarcinoma gene expression dataset, dataset constructed using low-rank prediction with 50% observability, and dataset constructed using sampling values with a uniform distribution at 50% observability. The table lists the results of sections of test instances in a cross-validation evaluation.

		Original		Low-rank prediction		Uniform distribution sampled	
Test Instance	Actual	Classifier predicted	Prob. dist	Classifier predicted	Prob. dist	Classifier predicted	Prob. dist
1	PDAC tumor	PDAC tumor	0.008, 0.992	PDAC tumor	0.001, 0.999	Normal pancreatic	0.978, 0.022
2	PDAC tumor	Normal pancreatic	0.555, 0.445	Normal pancreatic	0.997, 0.003	Normal pancreatic	0.62, 0.38
3	PDAC tumor	PDAC tumor	0.039, 0.961	Normal pancreatic	0.685, 0.315	PDAC tumor	0.48, 0.52
4	PDAC tumor	PDAC tumor	0.038, 0.962	PDAC tumor	0.091, 0.909	PDAC tumor	0.315, 0.685
5	Normal pancreatic	Normal pancreatic	0.962, 0.038	Normal pancreatic	1, 0	Normal pancreatic	0.663, 0.337
6	Normal pancreatic	Normal pancreatic	0.995, 0.005	Normal pancreatic	1, 0	PDAC tumor	0.164, 0.836
7	Normal pancreatic	Normal pancreatic	1, 0	Normal pancreatic	1, 0	Normal pancreatic	0.931, 0.069
8	Normal pancreatic	Normal pancreatic	1, 0	Normal pancreatic	1, 0	PDAC tumor	0.038, 0.962
9	PDAC tumor	PDAC tumor	0.001, 0.999	PDAC tumor	0, 1	Normal pancreatic	0.583, 0.417
10	PDAC tumor	Normal pancreatic	0.633, 0.367	PDAC tumor	0, 1	Normal pancreatic	0.591, 0.409
11	PDAC tumor	PDAC tumor	0, 1	PDAC tumor	0, 1	PDAC tumor	0.078, 0.922
12	PDAC tumor	Normal pancreatic	0.883, 0.117	Normal pancreatic	0.998, 0.002	PDAC tumor	0.13, 0.87
13	Normal pancreatic	Normal pancreatic	0.896, 0.104	Normal pancreatic	0.998, 0.002	Normal pancreatic	0.566, 0.434
14	Normal pancreatic	Normal pancreatic	1, 0	Normal pancreatic	0.992, 0.008	PDAC tumor	0.253, 0.747
15	Normal pancreatic	Normal pancreatic	0.955, 0.045	Normal pancreatic	1, 0	PDAC tumor	0.384, 0.616
16	Normal pancreatic	Normal pancreatic	0.871, 0.129	Normal pancreatic	0.883, 0.117	PDAC tumor	0.206, 0.794
17	PDAC tumor	PDAC tumor	0.002, 0.998	PDAC tumor	0.017, 0.983	Normal pancreatic	0.959, 0.041
18	PDAC tumor	PDAC tumor	0, 1	PDAC tumor	0.005, 0.995	PDAC tumor	0.279, 0.721
19	PDAC tumor	PDAC tumor	0.002, 0.998	Normal pancreatic	0.595, 0.405	Normal pancreatic	0.704, 0.296
20	PDAC tumor	PDAC tumor	0.02, 0.98	PDAC tumor	0.002, 0.998	PDAC tumor	0.442, 0.558
21	Normal pancreatic	Normal pancreatic	0.999, 0.001	Normal pancreatic	0.751, 0.249	PDAC tumor	0.442, 0.558
22	Normal pancreatic	Normal pancreatic	1, 0	Normal pancreatic	1, 0	PDAC tumor	0.442, 0.558
23	Normal pancreatic	PDAC tumor	0.0670.933	PDAC tumor	0.106, 0.894	PDAC tumor	0.279, 0.721
24	Normal pancreatic	Normal pancreatic	1, 0	Normal pancreatic	1, 0	PDAC tumor	0.442, 0.558
25	PDAC tumor	PDAC tumor	0, 1	PDAC tumor	0, 1	PDAC tumor	0.032, 0.968
26	PDAC tumor	PDAC tumor	0.004, 0.996	PDAC tumor	0, 1	PDAC tumor	0.104, 0.896
27	PDAC tumor	PDAC tumor	0, 1	PDAC tumor	0.028, 0.972	PDAC tumor	0.312, 0.688
28	PDAC tumor	PDAC tumor	0, 1	PDAC tumor	0.001, 0.999	Normal pancreatic	0.909, 0.091
29	Normal pancreatic	PDAC tumor	0, 1	PDAC tumor	0, 1	Normal pancreatic	0.74, 0.26
30	Normal pancreatic	Normal pancreatic	1, 0	Normal pancreatic	1, 0	Normal pancreatic	0.516, 0.484
31	Normal pancreatic	PDAC tumor	0.003, 0.997	Normal pancreatic	0.991, 0.009	Normal pancreatic	0.957, 0.043
32	Normal pancreatic	Normal pancreatic	1, 0	Normal pancreatic	0.965, 0.035	PDAC tumor	0.173, 0.827

Abbreviations: PDAC, Pancreatic ductal adenocarcinoma; Prob. dist, probability distribution.

References

Stirewalt, D. L., *et al*. (2008). Identification of genes with abnormal expression changes in acute myeloid leukemia. *Genes, Chromosomes and Cancer*, *47*(1), 8-20.

Lu, T. P., *et al.* (2010). Identification of a novel biomarker, SEMA5A, for non–small cell lung carcinoma in nonsmoking women. *Cancer Epidemiology Biomarkers & Prevention*, *19*(10), 2590-2597.

Boyle,J.O., *et al.* (2010). Effects of cigarette smoke on the human oral mucosal transcriptome. *Cancer Prevention Research*, ***3***(3), 266–278.

Badea, L., *et al.* (2008). Combined Gene Expression Analysis of Whole-Tissue and Microdissected Pancreatic Ductal Adenocarcinoma identifies Genes Specifically Overexpressed in Tumor Epithelia. *Hepato-gastroenterology*, *55*(88), 2016.
