# Supplementary material for: Gene expression prediction using low-rank matrix completion
Source: BMC Bioinformatics. 2016 Jun 17;17:243. doi: 10.1186/s12859-016-1106-6 (PMC4912738; doi:10.1186/s12859-016-1106-6)
Supplement: Additional file 2 — In this file, we provide description and sources of studies used in this study. (PDF 516 kb) [file 12859_2016_1106_MOESM2_ESM.pdf]

Additional File:

Gene Expression Prediction using Low-Rank Matrix Completion

Arnav Kapur[1,*] Kshitij Marwah[1] Gil Alterovitz[1,2]

[1]Biomedical Cybernetics Laboratory, Harvard Medical School, Boston, MA 02115 and [2]Department of Health Science and Technology/Electrical Engineering and Computer Science, Massachusetts Institute of Technology, Cambridge, MA 02139.

Authors	Description	Public Source
Bakay M, Wang Z, Melcon G, Schiltz L et al. & Dadgar S, Wang Z, Johnston H, Kesari A et al.	Analysis of muscle biopsy specimens from patients with various muscle diseases. Results provide insight into the diagnosis and pathogenesis of muscle diseases.	Link
Riester M, Taylor JM, Feifer A, Koppie T et al. & Riester M, Werner L, Bellmunt J, Selvarajah S et al.	Analysis of bladder cancer specimens from a high-risk population of patients who underwent radical cystectomy (Memorial Sloan-Kettering Cancer Center cohort). Results provide insight into the prediction of survival in high-risk urothelial carcinoma of the urinary bladder.	Link
Suresh R, Li X, Chiriac A, Goel K et al.	Analysis of circulating blood from first-time acute myocardial infarction (AMI) patients within 48 hours of MI. Results provide insight into molecular mechanisms underlying the response of circulating cells to first-time AMI.	Link
Yao Z, Jaeger JC, Ruzzo WL, Morale CZ et al.	Analysis of cultured skin fibroblasts prepared from patients with Marfan syndrome (MFS). MFS is a heritable connective tissue disorder caused by mutations in the fibrillin-1 gene. Results provide insight into the molecular pathogenesis of MFS.	Link
Maningat PD, Sen P, Rijnkels M, Hadsell DL et al.	Analysis of milk fat globule from milk samples collected from normal, lactating women every 3hr for 4 days (1 day of baseline/pre-rhGH administration and 3 days of high-dose rhGH administration). Results provide insight into potential molecular mechanisms for increasing milk production.	Link
Landi MT, Dracheva T, Rotunno M, Figueroa JD et al.	Analysis of different tumor stage adenocarcinoma and paired normal lung tissues of current, former and never smokers. To date, tobacco smoking is responsible for over 90% of lung cancers. Results provide insight into the molecular basis of lung carcinogenesis induced by smoking.	Link
Chen-Plotkin AS, Geser F, Plotkin JB, Clark CM et al.	Analysis of various brain regions of patients who suffered from frontotemporal lobar degeneration with ubiquitinated inclusions (FTLD-U). Presence of progranulin (GRN) mutations determined. GRN mutations are associated with FTLD-U. Results identify expression signatures for GRN subtypes of FTLD-U.	Link
Pei H, Li L, Fridley BL, Jenkins GD et al.	Analysis of tumor tissue and normal tissue in pancreatic cancer samples. The fresh frozen samples were obtained during surgical procedures. Results provide insight into molecular mechanisms underlying tumorigenesis.	Link
Yao Y, Richman L, Morehouse C, de los Reyes M et al.	Analysis of lesional and non-lesional skins from patients with psoriasis. Psoriasis is an immune-mediated disease characterized by aberrant epidermal differentiation, surface scale formation, and marked cutaneous inflammation. Results provide insight into the molecular pathogenesis of psoriasis.	Link
Miesner M, Haferlach C, Bacher U, Weiss T et al.	Analysis of BMMCs from untreated patients diagnosed as AML-MLD-sole (AML with myelodysplasia-related changes solely because of multilineage dysplasia) or AML-NOS (AML-not otherwise specified) according to WHO 2008 guidelines. Results provide insight into the relevance of MLD for AML classification.	Link
Stirewalt DL, Meshinchi S, Kopecky KJ, Fan W et al.	Comparison of leukemic blasts from 26 acute myeloid leukemia (AML) patients with normal hematopoietic cells at a variety of different stages of maturation from 38 healthy donors. Results provide insight into the possible clinical significance of those genes with AML-specific expression changes.	Link
Alter MD, Kharkar R, Ramsey KE, Craig DW et al.	Analysis of peripheral blood lymphocytes of autistic and non-autistic children. Correlation among gene expression changes, paternal age, and autism examined.	Link

Metzeler KH, Hummel M, Bloomfield CD, Spiekermann K et al.	Analysis of mononuclear cells from bone marrow or peripheral blood from a training set of 163 adult patients with cytogenetically normal acute myeloid leukemia (CN-AML). Patients with CN-AML show heterogeneous treatment outcomes. Results provide insight into a prognostic gene signature for CN-AML.	Link
Hatzis C, Sun H, Yao H, Hubbard RE et al.	Analysis of breast tumor samples preserved using 2 different RNA stabilization methods (RNAlater or snap freezing) up to 3 hours post tumor excision. Results provide insight into the effects of tissue handling on RNA integrity and microarray measurements from resected breast cancers.	Link
Pfister TD, Reinhold WC, Agama K, Gupta S et al. & Kohn KW, Zeeberg BM, Reinhold WC, Pommier Y.	Analysis of cell lines from 9 different cancer tissue of origin types (Breast, Central Nervous System, Colon, Leukemia, Melanoma, Non-Small Cell Lung, Ovarian, Prostate, and Renal) from the NCI-60 panel. Results provide insight into molecular mechanisms underlying the various cancer types.	Link
Iwamoto T, Bianchini G, Booser D, Qi Y et al.	Analysis of pre-treatment biopsies from 103 HER2-normal breast cancer patients (ER-positive and ER-negative subtypes) from MD Anderson Cancer Center/Institut Goustave Russy. Results provide insight into molecular mechanisms associated with chemotherapy sensitivity in breast cancer subtypes.	Link
Suárez-Fariñas M, Li K, Fuentes-Duculan J, Hayden K et al.	Analysis of 85 paired lesional and non-lesional samples from moderate-to-severe psoriasis patients at baseline without active psoriasis therapy. Results provide insight into the molecular mechanisms underlying psoriasis.	Link
Mura M, Anraku M, Yun Z, McRae K et al.	Analysis of lung from pulmonary fibrosis (PF) patients divided into different groups based on the mean pulmonary arterial pressure (mPAP): severe pulmonary hypertension (PH) group, intermediate PH group, NoPH group. Results provide insight into the pathobiology of PF with associated PH.	Link
Alhopuro P, Sammalkorpi H, Niittymäki I, Biström M et al.	Analysis of colorectal adenocarcinomas with microsatellite instability (MSI CRCs). MSI, caused by defects in the mismatch repair system, is observed in a subset of CRCs. Results provide insight into molecular mechanisms contributing to MSI CRC development.	Link
Dezso Z, Nikolsky Y, Sviridov E, Shi W et al.	Analysis of various normal tissues. Results provide insight into housekeeping and tissue-specific genes.	Link
Jans A, Konings E, Goossens GH, Bouwman FG et al.	Analysis of skeletal muscle (SM) from insulin resistant men 4 hrs after consumption of meals high in saturated FA (SFA), monounsaturated FA (MUFA), or polyunsaturated FA (PUFA). Results provide insight into mechanisms underlying effects of FA composition on SM FA handling and insulin sensitivity.	Link
Koh W, Pan W, Gawad C, Fan HC et al.	Analysis of cell-free plasma from pregnant women during the first, second, third trimesters and immediately post-partum. Results provide insight into a noninvasive means to monitor the expression status of many tissues and measure temporal expression of genes longitudinally during development.	Link
Coldren CD, Nick JA, Poch KR, Woolum MD et al.	Analysis of neutrophils that transmigrate to the alveolar space. Transmigration induced by bronchoscopic instillation of volunteers with endotoxin (LPS). Results identify differences between pulmonary and circulating neutrophils that occur early in endotoxin-induced lung inflammation.	Link
Dahia PL, Ross KN, Wright ME, Hayashida CY et al.	Analysis of 76 adrenal and extra-adrenal pheochromocytomas. These neural crest-derived tumors of uniform phenotype arise from inherited or sporadic mutations in at least six independent genes. Results provide insight into the molecular pathogenesis of pheochromocytomas.	Link
Kuner R, Muley T, Meister M, Ruschhaupt M et al.	Comparison of two non-small cell lung cancer histological subtypes: adenocarcinomas (AC) and squamous cell carcinomas (SCC). Results provide insight into the molecular differences between AC and SCC.	Link
Boersma BJ, Reimers M, Yi M, Ludwig JA et al. & Martin DN, Boersma BJ, Yi M, Reimers M et al.	Analysis of tumor epithelia from patients with inflammatory breast cancer (IBC) and invasive non-IBC. This is part of a study that also examines the expression profiles of stromata surrounding tumors. Results provide insight into the contribution of the tumor component to the pathogenesis of IBC.	Link
Ray M, Dharmarajan S, Freudenberg J, Zhang W et al.	Analysis of donor lung biopsies from recipients that developed primary graft dysfunction after lung transplantation. Results provide insight into the pathogenesis of primary lung graft dysfunction.	Link
Schulte A, Günther HS, Phillips HS, Kemming D et al & Günther HS, Schmidt NO, Phillips HS, Kemming D et al. & Zamykal M, Martens T, Matschke J, Günther HS et al.	Analysis of glioblastoma stem-like (GS) cell lines, corresponding glioblastoma primary tumors, conventional glioma cell lines, and GS neurospheres. Results provide insight into cell lines recapitulating transcriptional aspects of glioblastomas, thereby allowing identification of therapeutic targets.	Link

Parnell GP, Tang BM, Nalos M, Armstrong NJ et al.	Analysis of whole blood samples from survivors and non-survivors of sepsis for up to 5 days after their admission to the intensive care unit. Results provide insight into the molecular basis of the immune dysfunction resulting from sepsis.	Link
Wu Y, Grabsch H, Ivanova T, Tan IB et al. & Chia NY, Deng N, Das K, Huang D et al. & Lei Z, Tan IB, Das K, Deng N et al.	Analysis of 70 primary gastric tumors representing 3 subtypes (invasive, metabolic, and proliferative) from the Australian patient cohort (AU-2). Gastric adenocarcinomas show sizable heterogeneity between patients. Results provide insight into molecular characterization of gastric cancer subtypes.	Link
Metzeler KH, Hummel M, Bloomfield CD, Spiekermann K et al.	Analysis of mononuclear cells from bone marrow or peripheral blood from a training set of 163 adult patients with cytogenetically normal acute myeloid leukemia (CN-AML). Patients with CN-AML show heterogeneous treatment outcomes. Results provide insight into a prognostic gene signature for CN-AML.	Link
Shehata M, Demirtas D, Schnabl S, Hilgarth M et al.	Analysis of CD19+ selected B cells from CLL patients before and after chemoimmunotherapy regimens of rituximab (R), fludarabine and cyclophosphamide (FC), or RFC. Results provide insight into the molecular mechanisms underlying the beneficial effects of the chemoimmunotherapy regimens.	Link
Bouwens M, Grootte Bromhaar M, Jansen J, Müller M et al.	Analysis of peripheral blood mononuclear cells of individuals before and 6 hours after the consumption of shakes containing either mainly polyunsanturated (PUFA) or saturated fatty acids (SFA). Results provide insight into the molecular basis of the beneficial effect of PUFA on health.	Link
Gröne J, Lenze D, Jurinovic V, Hummel M et al.	Analysis of tumor cells from sporadic stage UICC II colon cancer patients who were treated by elective standard oncological resection but developed relapse during follow-up. Results provide insight into the challenges of constructing molecular signatures predictive for patient outcome.	Link
Boni JP, Leister C, Bender G, Fitzpatrick V et al.	Expression profiling of peripheral blood mononuclear cells (PBMC) from patients with advanced renal cancer following treatment with the rapamycin analog CCI-779. Gene expression examined 8 and 16 weeks after treatment. Results identify potential gene markers of CCI-779 exposure.	Link
Boyle JO, Gümüs ZH, Kacker A, Choksi VL et al.	Analysis of oral mucosae from 40 cigarette smokers and 40 age and gender matched never-smokers. Results provide insight into the carcinogenic effects of cigarette smoke.	Link
Bruchova H, Vasikova A, Merkerova M, Milcova A et al.	Analysis of placentas of women who smoked during pregnancy. Smoking increases the risk of preterm delivery and complications such as placental previa and abruption. Results provide insight into the molecular basis of smoke-induced placental abnormalities.	Link
Pilbrow AP, Folkersen L, Pearson JF, Brown CM et al.	Analysis of left ventricular heart from donors with no previous history of heart disease. Results provide insight into associations between 9p21.3 (rs1333049) genotype and transcripts in cardiovascular tissue.	Link
Johnson-Huang LM, Suárez-Fariñas M, Pierson KC, Fuentes-Duculan J et al.	Analysis of non-lesional (NL) skin of psoriasis patients 24 hrs after a single intradermal injection of interferon-γ (IFN-γ) to the NL skin. Results suggest that IFN-γ may prime an inflammatory environment in the skin that shares many features with psoriasis.	Link
Baty F, Facompré M, Wiegand J, Schwager J et al. et al.	Analysis of blood samples from 6 individuals at various time points up to 12 hours following the intake of water, ethanol, grape juice, or red wine. Results may contribute to elucidating the mechanisms underlying the cardioprotective effects of red wine.	Link
Clelland CL, Read LL, Panek LJ, Nadrich RH et al.	Analysis of peripheral blood leukocytes from first-episode, never-medicated bipolar disorder (BPD) patients and medicated BPD patients. Results provide insight into a peripheral biomarker profile for BPD that is not induced by medication.	Link
Orsmark-Pietras C, James A, Konradsen JR, Nordlund B et al. & Acevedo N, Reinius LE, Greco D, Gref A et al.	Analysis of white blood cells from children with severe therapy-resistant asthma and those with controlled persistent mild asthma. Results provide insight into the molecular pathogenesis of these asthma phenotypes.	Link
Johnson JM, Castle J, Garrett-Engele P, Kan Z et al.	Monitoring of mRNA splice variants for more than 10,000 multi-exon genes using arrays with oligonucleotide probes positioned at exon-exon junctions. mRNA from 52 tissues and cell lines examined. Results provide tissue distribution of splice variants and identify novel splice variants.	Link
Votavova H, Dostalova Merkerova M, Fejglova K, Vasikova A et al.	Analysis of peripheral blood leukocytes and placenta of pregnant smokers. Umbilical cord blood leukocytes of their newborns also examined. Maternal smoking has a negative effect on all stages of pregnancy. Results provide insight into molecular mechanisms underlying tobacco smoke-related defects.	Link
Zhang G, Schetter A, He P, Funamizu N et al. & Zhang G, He P, Tan H, Budhu A et al.	Analysis of 45 matching pairs of pancreatic ductal adenocarcinoma (PDAC) tumor and adjacent non-tumor tissue. Results provide insight into tumor markers with prognostic significance and molecular mechanisms underlying PDAC.	Link

Wu X, Wang J, Cui X, Maianu L et al. & Wu X, Patki A, Lara-Castro C, Cui X et al.	Analysis of vastus lateralis muscle biopsies from insulin-sensitive subjects, insulin-resistant subjects, and diabetic patients, following insulin treatment. Results provide insight into the molecular basis of insulin action in skeletal muscle and the underlying defects causing insulin resistance.	Link
Casey T, Bond J, Tighe S, Hunter T et al.	Analysis of epithelium and stroma cells in normal and invasive breast cancer tissues. Information on the role that stroma tissues have on the growth and progression of cancer is limited. Results provide insights into the molecular basis of cancer invasion and metastasis.	Link
Farmer P, Bonnefoi H, Becette V, Tubiana-Hulin M et al.	Analysis of tumors of 49 breast cancer patients. Tumors classified into luminal and basal classes, and a novel molecular apocrine class. Apocrine tumors are estrogen receptor negative (ER-) and androgen receptor positive (AR+), while luminal tumors are ER+ and AR+, and basal tumors are ER- and AR-.	Link
Saligan LN, Hsiao CP, Wang D, Wang XM et al.	Analysis of peripheral blood from men with non-metastatic prostate cancer (PC) on days 1, 7, 14, 21, 42 of external beam radiation therapy (EBRT), and at 30 days post-EBRT. Fatigue is a common side effect of EBRT. Results provide insight into molecular basis of fatigue in men with PC during EBRT.	Link
Song MO, Li J, Freedman JH.	Analysis of HepG2 liver cells treated with up to 600 uM copper sulfate for up to 24 hours. Copper is an essential trace element but can be extremely toxic at supraphysiological levels. Results provide insight into the molecular basis of copper toxicity.	Link
Tsukamoto S, Ishikawa T, Iida S, Ishiguro M et al.	Analysis of LCM-isolated colorectal cancer (CRC) tumors representing various stages and metastases. Results provide insight into predictive biomarkers of metastasis and treatment targets in CRC.	Link
LaBreche HG, Nevins JR, Huang E.	Analysis of PBMCs from breast cancer patients, patients with benign breast abnormalities, healthy individuals, and patients with other types of cancer (GI, brain). These results, together with results from a murine breast cancer model, provide insight into developing a human breast tumor predictor.	Link
Iwamoto K, Kakiuchi C, Bundo M, Ikeda K et al.	Analysis of postmortem prefrontal cortices from subjects with bipolar disorder, depression, and schizophrenia. Results provide insight into the molecular pathophysiology of these mental disorders.	Link
Freije WA, Castro-Vargas FE, Fang Z, Horvath S et al.	Analysis of grades III and IV gliomas of various histologic types. Results used to develop a gene-expression based, histology independent-classification scheme, and provide insight into the biology of gliomas.	Link
Bannon MJ, Johnson MM, Michelhaugh SK, Hartley ZJ et al.	Analysis of postmortem midbrain specimens from individuals who died from cocaine abuse. Midbrain dopamine-synthesizing neurons play a important role in the addiction process. Results provide insight into the molecular pathophysiological changes in the midbrain associated with cocaine abuse.	Link
Pan F, Yang TL, Chen XD, Chen Y et al.	Analysis of peripheral circulating B cells from smoking and non-smoking healthy US white females. B cells are directly associated with the onset and development of many smoking-induced diseases. Results provide insight into the molecular basis of B cell involvement in smoking-related pathogenesis.	Link
Pellagatti A, Cazzola M, Giagounidis A, Perry J et al.	Analysis of bone marrow CD34+ hematopoietic stem cells of myelodysplastic syndrome (MDS) patients. MDS is a group of clonal hematopoietic stem cell malignancies characterized by ineffective hematopoiesis. Results provide insight into the molecular pathogenesis of MDS.	Link
Richardson AL, Wang ZC, De Nicolo A, Lu X et al. & Alimonti A, Carracedo A, Clohessy JG, Trotman LC et al.	Analysis of sporadic basal-like cancer (BLC), BRCA-associated breast cancer, and non-BLC tumors. Sporadic BLC are phenotypically similar to BRCA1-associated cancers. Results provide insight into the molecular pathogenesis of BLC and BRCA1-associated breast cancer.	Link
Lu TP, Tsai MH, Lee JM, Hsu CP et al.	Analysis of paired tumor and adjacent normal lung tissue specimens obtained from nonsmoking female non-small cell lung carcinoma (NSCLC) patients in Taiwan. Results provide insight into potential prognostic biomarkers and therapeutic targets for NSCLC.	Link
Uronis JM, Osada T, McCall S, Yang XY et al.	Analysis of patient-derived colorectal cancer explants (PDCCE), generated by direct transplantation of human colorectal cancer (CRC) tissues into NOD-SCID mice, and matched patient CRC primary tumors. Results provide insight into the degree to which PDCCEs represent their primary human counterparts.	Link
Spira A, Beane JE, Shah V, Steiling K et al. & Gustafson AM, Soldi R, Anderlind C, Scholand MB et al.	Analysis of large airway epithelial cells from cigarette smokers without cancer, with cancer, and with suspect lung cancer. Results provide insight into the feasibility of using gene expression to detect early stage lung cancer in smokers.	Link

Brune V, Tiacci E, Pfeil I, Döring C et al & Giefing M, Winoto-Morbach S, Sosna J, Döring C et al.	Analysis of neoplastic lymphocytic and histiocytic cells (L&H) dissected from nodular lymphocyte-predominant Hodgkin lymphoma tumors (NLPHL). L&H cells represent less than 1 percent of cells in NLPHL tumors. Results provide insight into the pathogenesis of NLPHL.	Link
Jorgensen E, Stinson A, Shan L, Yang J et al.	Analysis of normal bronchial epithelial cells exposed to cigarette smoke from a typical light flavor brand for up to 24 hours. Results provide insight into the impact of cigarette smoke exposure at the molecular level.	Link
Rajaram M, Li J, Egeblad M, Powers RS.	Analysis of fibroblasts (HFF1, HFF2, Wi38, CCD1112Sk) and breast cancer epithelial cells (Cal51, MDA-MB-231) FACS-sorted from 6-day cocultures. Cancer-associated fibroblasts can promote tumor formation and growth. Results provide insight into tumor-fibroblast interactions involved in tumorigenicity.	Link
Kimbung S, Kovács A, Bendahl PO, Malmström P et al.	Analysis of breast cancer (BC) metastases from different anatomical sites collected prior to treatment. The anatomical location of the metastatic lesion is associated with length of survival post-recurrence. Results provide insight into molecular mechanisms underlying BC site-specific metastases.	Link
Boersma BJ, Reimers M, Yi M, Ludwig JA et al. & Martin DN, Boersma BJ, Yi M, Reimers M et al.	Analysis of stromata surrounding tumors from patients with inflammatory breast cancer (IBC) and invasive non-IBC. This is part of a study that also examines the expression profiles of tumors. Results provide insight into the contribution of the tumor stroma component to the pathogenesis of IBC.	Link
Parnell G, McLean A, Booth D, Huang S et al.	Analysis of whole blood (WB) from critically ill influenza and bacterial pneumonia patients for up to 5 days, and WB from before and 7 days post-influenza vaccination volunteers. Results provide insight into the role of host response in influencing disease progression in influenza infection.	Link
Robinson G, Parker M, Kranenburg TA, Lu C et al.	Analysis of medulloblastomas from children ages 3 to 16 years. Medulloblastoma is a malignant childhood brain tumor comprising four discrete subgroups. Results provide insights into pathogenesis of medulloblastoma and highlight targets for therapeutic development.	Link
Lu NZ, Collins JB, Grissom SF, Cidlowski JA. & Jewell CM, Scoltock AB, Hamel BL, Yudt MR et al.	Analysis of glucocorticoid receptor alpha (hGRalpha) isoform -A, -B, -C, or -D expressing osteosarcoma cells for up to 24 h after hGRalpha activation with dexomethasone. Cell apoptosis occurred in a GR isoform-selective manner. Results provide insight into the function of each isoform.	Link
Rieger KE, Hong WJ, Tusher VG, Tang J et al.	Analysis of UV and IR irradiated lymphoblastoid cell lines derived from the peripheral blood of patients with acute radiation toxicity. Samples taken 2 months after the completion of radiation therapy. Results provide insight into the role of the response to DNA damage in radiation toxicity.	Link
Bakay M, Wang Z, Melcon G, Schiltz L et al. & Dadgar S, Wang Z, Johnston H, Kesari A et al.	Analysis of muscle biopsy specimens from patients with various muscle diseases. Results provide insight into the diagnosis and pathogenesis of muscle diseases.	Link
Liu PT, Stenger S, Li H, Wenzel L et al.	Analysis of monocytes (MOs) and MO-derived dendritic cells (DCs) for up to 24 hours following Mycobacterium tuberculosis-derived lipopeptide treatment. Toll-like receptor activation by bacterial lipopeptides reduced the viability of M. tuberculosis in MOs and macrophages but not MO-derived DCs.	Link
Rahimov F, King OD, Leung DG, Bibat GM et al.	Analysis of biceps and deltoids of facioscapulohumeral muscular dystrophy (FSHD) subjects and their unaffected first-degree relatives. FSHD is progressive neuromuscular disorder affecting biceps more severely than deltoid muscles. Results provide insight into molecular basis of FSHD pathogenesis.	Link
Ye QH, Qin LX, Forgues M, He P et al.	Generation of a molecular signature for metastatic hepatocellular carcinoma (HCC) and identification of genes relevant to metastasis and patient survival. Osteopontin identified as molecular marker for defining metastatic potential.	Link
Miesner M, Haferlach C, Bacher U, Weiss T et al.	Analysis of BMMCs from untreated patients with AML-MRC (AML with myelodysplasia-related changes) and from the combined group AML-NOS plus AML-MLD-sole on the basis of cytogenetics or a myelodysplastic syndrome (MDS) history. Results provide insight into the relevance of MLD for AML classification.	Link
Dürig J, Dührsen U, Klein-Hitpass L, Worm J et al.	Temporal analysis of peripheral blood from 18 relapsed CLL patients treated with a maximum of 6 doses (0.2-6mg/kg) of SPC2996, an antisense molecule targeting the mRNA of Bcl-2 oncoprotein. Results provide insight into the molecular basis of the immunostimulatory effects of SPC2996 in CLL.	Link
Liu NW, Sanford T, Srinivasan R, Liu JL et al.	Analysis of ten renal tumors that were resected then immediately frozen or stored at 4°C, 22°C, 37°C for up to 240 minutes. Results provide insight into the impact of tissue acquisition method on tumor gene expression profiles.	Link

Ryan MM, Lockstone HE, Huffaker SJ, Wayland MT et al.	Analysis of postmortem dorsolateral prefrontal cortex from 30 adults with bipolar disorder. Results provide insight into the pathophysiology of the disease.	Link
Newell KA, Asare A, Kirk AD, Gisler TD et al.	Analysis of whole blood from tolerant renal transplant recipients (i.e., with stable graft function and receiving no immunosuppressive drugs for >1 year) and from recipients stable on immunosuppressive drugs. Results provide insight into molecular mechanisms underlying renal transplant tolerance.	Link
Heap GA, Trynka G, Jansen RC, Bruinenberg M et al.	Expression analysis of untouched primary leukocytes from unrelated celiac disease individuals. Results used in conjunction with genome-wide association genotype data provide insight into genetic variation effects on gene expression in primary leukocytes from an immune-mediated disease.	Link
Kwissa M, Nakaya HI, Onlamoon N, Wrammert J et al.	Analysis of blood from patients with acute Dengue virus (DENV) infection and during convalescence. Dengue is a mosquito-borne infectious disease and Dengue Hemorrhagic Fever is a life-threatening illness. Results provide insight into molecular mechanisms underlying host response to DENV infection.	Link
Dombroski BA, Nayak RR, Ewens KG, Ankener W et al.	Analysis of immortalized B cells from 60 unrelated individuals (grandparents in the HapMap CEPH-Utah pedigrees) treated in vitro with tunicamycin to induce ER stress. ER stress induces the unfolded protein response (UPR). Results provide insight into molecular mechanisms underlying UPR response.	Link
Eskandarpour M, Huang F, Reeves KA, Clark E et al.	Analysis of melanoma 224 and BL cells for up to 3 days after suppression of the mutant NRAS Q61R gene with siRNA siMut10 or siMut12. Activating mutations in NRAS gene is a common genetic event in malignant melanoma. Results provide insight into the role of NRAS in the pathogenesis of melanoma.	Link
Campo Dell'Orto M, Zangrando A, Trentin L, Li R et al.	Analysis of RNA samples prepared from leukemic white blood cells using 3 different protocols. Samples obtained from 27 pediatric patients with various subtypes of acute leukemia. Results provide insight into the impact of RNA preparation methods on the variation in gene expression data.	Link
Koth LL, Solberg OD, Peng JC, Bhakta NR et al. & Su R, Li MM, Bhakta NR, Solberg OD et al.	Analysis of whole blood from sarcoidosis and hypersensitivity pneumonitis patients. Results provide insight into molecular mechanisms underlying sarcoidosis and hypersensitivity pneumonitis in peripheral blood.	Link
Agnelli L, Bicciato S, Mattioli M, Fabris S et al.	Analysis of CD138+ plasma cells purified from bone marrow of multiple myeloma (MM) patients. Results used to classify MM cases into translocation/cyclin (TC) groups based on cyclin D expression and presence of translocations in the immunoglobulin heavy chain locus at 14q32.	Link
Papapanou PN, Sedaghatfar MH, Demmer RT, Wolf DL et al.	Analysis of peripheral blood monocytes from periodontitis patients up to 10 weeks after initiation of periodontal therapy. Periodontitis is associated with the process of atherosclerosis. Results provide insight into the molecular basis of a systemic anti-inflammatory effect of periodontal therapy.	Link
Singh D, Fox SM, Tal-Singer R, Plumb J et al.	Analysis of sputum from ex-smokers with moderate (stage 2) or severe (stages 3, 4) chronic obstructive pulmonary disease (COPD). Induced sputum contains inflammatory cells, predominantly neutrophils and macrophages. Results provide insight into molecular mechanisms underlying COPD progression.	Link
Moran LB, Duke DC, Deprez M, Dexter DT et al.	Analysis of medial and lateral substantia nigras (SNs) from post-mortem brain samples obtained from individuals with sporadic Parkinson's disease (PD). The SN exhibits extensive tissue damage in PD. Results provide insight into the pathogenesis of PD.	Link
Taneera J, Lang S, Sharma A, Fadista J et al. &Taneera J, Fadista J, Ahlqvist E, Zhang M et al.	Analysis of pancreatic islets from type 2 diabetes (T2D) and non-diabetic cadaver donors. Glycemic control (HbA1c) levels also measured from the same individuals (normoglycemic: HbA1c < 6%; hyperglycemic: HbA1c ≥ 6%). Results provided insight into molecular basis of islet dysfunction in T2D.	Link
Gutierrez A Jr, Tschumper RC, Wu X, Shanafelt TD et al.	Analysis of peripheral blood B cells isolated from 41 untreated chronic lymphocytic leukemia (CLL) patients and 11 age-matched control subjects. B-cell CLL is the most common type of leukemia, mainly affecting adults. Results provide insight into molecular mechanisms underlying CLL pathogenesis.	Link
Wang L, Hurley DG, Watkins W, Araki H et al.	Analysis of cultured A375 melanoma cells depleted for various transcription factors and signaling proteins. Results provide insight into the molecular pathogenesis of melanoma.	Link
Tsuji S, Midorikawa Y, Takahashi T, Yagi K et al.	Analysis of primary or metastatic lesions from patients with unresectable colorectal cancer (CRC) prior to FOLFOX6 therapy (oxaliplatin, 5-fluorouracil, leucovorin regimen). Results provide insight into molecular signatures that predict responders to FOLFOX therapy for unresectable CRC.	Link

Ahrens M, Ammerpohl O, von Schönfels W, Kolarova J et al.	Analysis of liver from morbidly obese patient representing nonalcoholic fatty liver disease (NAFLD) subtypes steatosis and nonalcoholic steatohepatitis (NASH), post-bariatric surgery. Results provide insight into molecular basis of the NAFLD liver phenotypes and into postbariatric molecular changes.	Link
Ayari H, Bricca G.	Analysis of carotid artery atheromatous plaques from hypertensive patients. Atheromas are fatty deposits in the walls of arteries. Results provide insight into the molecular pathogenesis of atheroma.	Link
Zhang J, Ding L, Holmfeldt L, Wu G et al. & Gutierrez A, Kentsis A, Sanda T, Holmfeldt L et al.	Analysis of tumor cells from pediatric patients with early T-cell precursor acute lymphoblastic leukemia (ETP ALL). The ETP ALL subtype has a poor prognosis when treated with standard chemotherapy. Results provide insight into the molecular mechanisms underlying ETP ALL.	Link
Cole SW, Hawkley LC, Arevalo JM, Cacioppo JT.	Analysis of PBMCs from chronically lonely, older adults. Lack of close social ties is a well-established risk factor for diseases involving the immune system and inflammation. Results provide insight into the molecular mechanisms underlying those effects.	Link
Dyrskjøt L, Kruhøffer M, Thykjaer T, Marcussen N et al.	Analysis of bladder biopsies of superficial transitional cell carcinomas with or without surrounding carcinoma in situ (CIS) lesions and muscle invasive carcinomas (mTCC). CIS is a common mTCC precursor. Results provide insight into which tumors in early stage bladder cancer are likely to progress.	Link
Sun Y, Goodison S.	Analysis of prostate cancer primary tumors of known disease recurrence status. Results provide insight into the molecular mechanisms underlying recurrent and non-recurrent prostate cancer primary tumors.	Link
Iwamoto T, Bianchini G, Booser D, Qi Y et al.	Analysis of pre-treatment biopsies from 62 HER2-normal breast cancer patients (ER-positive and ER-negative subtypes) from US Oncology clinical trial 02103. Results provide insight into molecular mechanisms associated with chemotherapy sensitivity in breast cancer subtypes.	Link
Schlicker A, Beran G, Chresta CM, McWalter G et al.	Analysis of primary colorectal cancer (CRC) tumors. CRC is a heterogeneous disease. Results provide insight into stratifying CRC tumor samples into subtypes and tailoring treatments for the CRC subtypes.	Link
Pellagatti A, Cazzola M, Giagounidis AA, Malcovati L et al.	Analysis of CD34+ cells from 55 patients with myelodysplastic syndromes (MDSs). MDSs are a heterogeneous group of hematopoietic malignancies, characterized by blood cytopenias, ineffective hematopoiesis, and a hypercellular bone marrow. Results provide insight into the pathophysiology of MDS.	Link
Metzeler KH, Hummel M, Bloomfield CD, Spiekermann K et al.	Analysis of mononuclear cells from bone marrow or peripheral blood from a test set of adult patients with cytogenetically normal acute myeloid leukemia (CN-AML). CN-AML patients show heterogeneous treatment outcomes. Results provide insight into the prognostic value of a gene signature for CN-AML.	Link
Badea L, Herlea V, Dima SO, Dumitrascu T et al.	Analysis of pancreatic ductal adenocarcinoma (PDAC) tumors and matching normal pancreatic tissue from ICF pancreatic cancer patients. PDAC is an aggressive, rapidly disseminating, desmoplastic cancer with poor prognosis. Results provide insight into molecular processes involved in PDAC pathology.	Link
Schirmer SH, Fledderus JO, van der Laan AM, van der Pouw-Kraan TC et al.	Analysis of various mononuclear cells from patients with severe triple-vessel coronary artery disease (CAD). CD34+ stem cells, CD4+ T-helper cells, CD14+ resting monocytes, LPS-stimulated monocytes, and macrophages were examined. Results provide insight into the pathophysiology of atherosclerosis.	Link
Ockenhouse CF, Bernstein WB, Wang Z, Vahey MT.	Analysis of peripheral blood mononuclear cells from HIV-1 seropositive and seronegative individuals. Results used to identify a 10-gene signature used for determining HIV-1 serostatus, and a 6-gene signature to identify seropositive individuals exhibiting changes in CD4+ T cell counts.	Link
Tang BM, McLean AS, Dawes IW, Huang SJ et al.	Analysis of neutrophils from critically ill patients with sepsis. Results used to define a gene expression signature for sepsis and provides insight into the host response to sepsis.	Link
López-Corral L, Corchete LA, Sarasquete ME, Mateos MV et al.	Analysis of plasma cells from patients with monoclonal gammopathy of undetermined significance (MGUS), smoldering multiple myeloma (MM), or MM. MM is consistently preceded by MGUS/SMM. Results provide insight into molecular mechanisms underlying the transformation from precursor disease to MM.	Link
Jelinsky SA, Rodeo SA, Li J, Gulotta LV et al.	Analysis of diseased tendons from patients with tendinopathies. Tendinopathies are chronic tendon injuries, common conditions in professional and recreational athletes, as well as individuals engaging in repetitive activities. Results provide insight into the molecular pathogenesis of tendinopathy.	Link

Reis PP, Waldron L, Perez-Ordonez B, Pintilie M et al.	Analysis of oral carcinoma, histologically normal margins, and adjacent normal tissues from patients with squamous cell carcinoma (OSCC) of the tongue (training set). Results provide insight into molecular signature in histologically normal margins that is predictive of oral carcinoma recurrence.	Link
Phillips HS, Kharbanda S, Chen R, Forrest WF et al. ', 'Costa BM, Smith JS, Chen Y, Chen J et al.	Analysis of high-grade glioma (HGGs) samples from cases of WHO grade III and IV astrocytomas. Results classify HGGs into molecular subclasses with prognostic value that predict survival and disease progression. The molecular subclasses resemble key stages of neurogenesis.	Link
Gutiérrez NC, Ocio EM, de Las Rivas J, Maiso P et al.	Analysis of B lymphocytes (BL) and plasma cells (PC) from patients with Waldenstrom's macroglobulinemia (WM), a B-lymphoproliferative disorder (BLPD). Results provide insight into differences between PC and BL from WM and their cell counterpart in chronic lymphocytic leukemia and multiple myeloma.	Link
Steidl C, Lee T, Shah SP, Farinha P et al.	Analysis of diagnostic lymph-node biopsies from classic Hodgkins lymphoma HIV- patients before ABVD chemotherapy. Results identify gene expression signatures that correlate with treatment outcomes.	Link
Gelman BB, Chen T, Lisinicchia JG, Soukup VM et al.	Analysis of basal ganglia, frontal cortex, and white matter from HIV patients with HIV-associated dementia (HAD) or HAD plus HIV encephalitis (HIVE). Results provide insight into molecular events of neurological impairment and inflammation associated with HIV infection in different brain regions.	Link
Hokama M, Oka S, Leon J, Ninomiya T et al.	Analysis of postmortem brain tissues (frontal cortex, temporal cortex, hippocampus) from male and female Hisayama residents pathologically diagnosed as having Alzheimer's disease (AD) or an AD-like disorder. Results provide insight into the molecular mechanisms underlying AD brain pathology.	Link
Doering TA, Crawford A, Angelosanto JM, Paley MA et al.	Analysis of CD4+ and CD8+ T cells responding to LCMV-Armstrong or LCMV-Clone 13.	Link
Xu X, Gnatenko DV, Ju J, Hitchcock IS et al	Analysis of microRNA fingerprints in thrombocythemic platelets using expression patterns in 79 subjects with thrombocytosis and healthy controls, and integrated these data with transcriptomic and proteomic platforms.	Link
Desmedt C, Majjaj S, Kheddoumi N, Singhal SK et al	Characterization and clinical evaluation of CD10+ stroma cells in the breast cancer microenvironment.	Link
Forde N, Carter F, Spencer TE, Bazer FW et al	Effects of pregnancy and progesterone supplementation on endometrial gene expression in cattle.	Link
Korzelius J, Naumann SK et al.	RNA-Seq of amplified mRNA from sorted esg+-midgut progenitor cells expressing esgRNAi or UAS-esg AND RNA-Seq of whole midgut tissue from flies overexpressing UAS-esg and UAS-esg+UAS-dIAP (+control empty vector RNAi, UAS-dIAP and w1118) using the MyoIA-Gal4ts EC-driver.	Link
Goolam M, Scialdone A et al.	Heterogeneity in Oct4 and Sox2 Targets Biases Cell Fate in Four-Cell Mouse Embryos.	Link
Scialdone A, Natarajan KN et al.	Computational assignment of cell-cycle stage from single-cell transcriptome data.	Link
Rinaldi A, Barone I	Analysis of the clinical significance of its PDE5 in breast cancers and the underlying molecular mechanisms by which it may contribute to breast cancer progression.	Link
Stubbington MJT, Lönnberg T et al.	Single-cell RNA-seq from CD4+ T lymphocytes from uninfected steady-state mouse, two mice with Salmonella typhimurium infection at day 14 and one mouse at day 49 post-infection. Used to demonstrate application of reconstruction and analysis of T cell receptor sequences from single-cell RNA-seq.	Link
Slabbinck B, InzĂŠ D et al.	Analysis of 94 RILs of a maize multiparental advanced generation intercross (MAGIC) population, originating from nine parental lines (A632, B73, B96, F7, H99, HP301, Mo17, W153R and CML91) followed by 6 generations of self-pollination. A subset of 94 lines was chosen randomly from the set of 529 lines that was genotyped and phenotyped in the field (Dell'Aqua et al (2015) Genome Biology, 16:167) and sampled for RNA seq of proliferative tissue of growing leaf.	Link
Liu J	RNAseq data to study PRPF6 regulated splice forms in colon cancer cell lines.	Link
Thomas G, Ruckerl D et al.	Transcription profiling by high throughput sequencing of macrophages obtained from BALB/c and IL4Ra-/- mice via thioglycollate-elicitation or Brugia malayi implantation.	Link
Goncalves A, Leigh-Brown S et al.	RNA-seq of liver samples from C57BL/6J, CAST/EiJ mice as well as their initial and reciprocal F1 progeny to study the compensatory cis-trans regulation of liver gene expression.	Link
Kommadath A, Bao H et al. & Bao H, Kommadath A et al.	RNA-seq of coding RNA from whole blood samples of pigs before and after Salmonella enterica serovar Typhimurium challenge and with different levels of faecal Salmonella shedding. The study	Link

	aimed to identify porcine genes and gene co-expression networks that differentiate distinct responses to Salmonella challenge with respect to faecal Salmonella shedding.	
Sieker JT, Ayturk UM et al.	Analysis of the effects of corticosteroid treatment using RNA-seq of synovial membranes from a preclinical model of post-traumatic knee osteoarthritis.	Link
Gutteridge A	Transcriptional and epigenetic profiling of EP-derived iPSCs (RNASeq).	Link
Fagerberg L, Hallström BJ et al.	RNA-seq of coding RNA from tissue samples of 95 human individuals representing 27 different tissues in order to determine tissue-specificity of all protein-coding genes.	Link
Wang C, Gu Y et al.	This study involved gene expression patterns of 24 lung adenocarcinoma patients from China to study the alteration of expression pattern of cancer-testis genes by the use of whole transcriptome sequencing.	Link
